# Reconstruction and *in vivo* analysis of the extinct *tbx5* gene from ancient wingless moa (Aves: Dinornithiformes)

**DOI:** 10.1186/1471-2148-14-75

**Published:** 2014-05-14

**Authors:** Leon Huynen, Takayuki Suzuki, Toshihiko Ogura, Yusuke Watanabe, Craig D Millar, Michael Hofreiter, Craig Smith, Sara Mirmoeini, David M Lambert

**Affiliations:** 1 Environmental Futures Centre, Griffith University, 170 Kessels Road, Nathan Qld 4111, Australia; 2 Division of Biological Science, Nagoya University, Nagoya 464-8602, Japan; 3 Institute of Development, Aging and Cancer (IDAC), Tohoku University, Sendai 980-8575, Japan; 4 Allan Wilson Centre for Molecular Ecology and Evolution, School of Biological Sciences, University of Auckland, Private Bag 92019, Auckland, New Zealand; 5 Department of Biology, University of York, York YO10 5DD, UK; 6 Faculty of Natural Sciences, University of Potsdam, 14476 Potsdam, Germany; 7 Murdoch Children’s Research Institute, Royal Children’s Hospital, Flemington rd Parkville, Victoria 3052, Australia; 8 Institute of Natural Sciences, Massey University, Auckland 0632, New Zealand

**Keywords:** tbx5, Moa, Gene expression, Ancient DNA, Development, Forelimb

## Abstract

**Background:**

The forelimb-specific gene *tbx5* is highly conserved and essential for the development of forelimbs in zebrafish, mice, and humans. Amongst birds, a single order, Dinornithiformes, comprising the extinct wingless moa of New Zealand, are unique in having no skeletal evidence of forelimb-like structures.

**Results:**

To determine the sequence of *tbx5* in moa, we used a range of PCR-based techniques on ancient DNA to retrieve all nine *tbx5* exons and splice sites from the giant moa, *Dinornis*. Moa Tbx5 is identical to chicken Tbx5 in being able to activate the downstream promotors of *fgf10* and *ANF*. In addition we show that missexpression of moa *tbx5* in the hindlimb of chicken embryos results in the formation of forelimb features, suggesting that Tbx5 was fully functional in wingless moa. An alternatively spliced exon 1 for *tbx5* that is expressed specifically in the forelimb region was shown to be almost identical between moa and ostrich, suggesting that, as well as being fully functional, *tbx5* is likely to have been expressed normally in moa since divergence from their flighted ancestors, approximately 60 mya.

**Conclusions:**

The results suggests that, as in mice, moa *tbx5* is necessary for the induction of forelimbs, but is not sufficient for their outgrowth. Moa Tbx5 may have played an important role in the development of moa’s remnant forelimb girdle, and may be required for the formation of this structure. Our results further show that genetic changes affecting genes other than *tbx5* must be responsible for the complete loss of forelimbs in moa.

## Background

**Figure 1:**
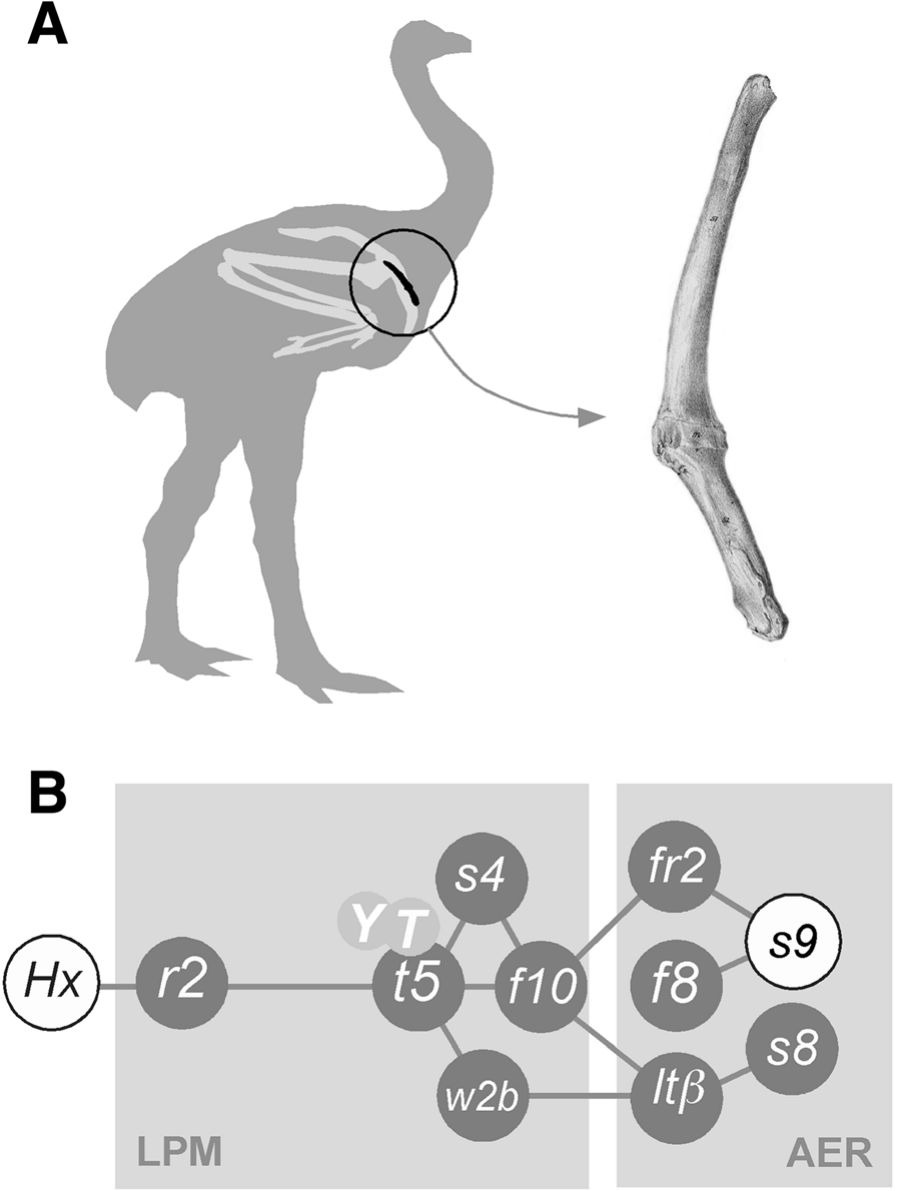
**Moa forelimb structure and forelimb initiation gene network. A**. The moa wing skeleture consists of a small finger-sized bone that represents the fusion of remnants of the coracoid and scapula to form the scapulocoracoid (right). A fully developed wing skeleton (light grey) is shown superimposed onto a moa sillouette for comparison. The scapulocoracoid is circled and shown in black. **B**. The central gene network essential for forelimb initiation. A Hox expression code initiates the expression of retinaldehyde dehydrogenase 2 (*r2*) and *tbx5* (*t5*) in the lateral plate mesoderm (LPM) [41]. Tbx5, in combination with YAP and TAZ (Y, T; [50]), interacts with Sall4 (*s4*) [42] to activate *fgf10* (*f10*) and subsequently *fgf8* (*f8*) in the limb apical ectodermal ridge (AER). An additional activator of forelimb initiation Wnt2b (*W2b*) possibly acts upstream of, or in conjunction with *tbx5* through the transcription activators LEF/TCF and beta-catenin (*ltβ*) [3,51].

Limb loss by regressive evolution is common in nature with well-known examples being whales, some salamanders, and snakes. Amongst birds, the loss of forelimbs is best represented amongst ratites, with a general reduction in wing size evident in the rhea and ostrich, and a more severe truncation of wings shown in emu, cassowary, kiwi, and elephant bird. Surprisingly, phylogenetically, New Zealand’s moa group with the fully winged and flighted tinamou [1], despite having lost almost all skeletal elements associated with the wing. The only evidence that moa once had wings is the presence of a small finger-sized bone, the scapulocoracoid, a fused remnant of the forelimb girdle’s coracoid and scapula (Figure 1A, B).

To determine the molecular basis of winglessness in moa, we present the first reconstruction and characterization of an ancient multi-exon gene, *tbx5*, a highly conserved Tbox motif-containing transcription factor known to be essential for forelimb initiation in nearly all animals. Tbx5 is an essential member of a tightly regulated gene network that includes the *Hox* genes (reviewed in [2]), *fgf8*[3], *fgf10*[4-6], ANF [7], and *sall4*[8]. Of these, *tbx5* is perhaps the best characterized [5,6,8-10]. Knockouts of *tbx5* in mice, or morpholino-induced *tbx5* knockdowns in zebrafish, result in the complete loss of forelimbs [11,12]. Similarly, natural occurrences of limblessness in humans and zebrafish have been attributed to mutations in *tbx5*. In humans, most *tbx5* mutations are found in the highly conserved DNA-binding Tbox region and result in Holt-Oram syndrome (HOS), characterized by reduction of the forelimbs and associated heart anomalies [9]. A similar phenotype is shown by the zebrafish *tbx5* mutant *heartstrings*[13].

The spectrum of mutations in Tbx5 that contribute to Holt-Oram syndrome continues to grow [9,14,15]. Tbx5 mutations have now been found in more than 70% of patients with a strict clinical diagnosis of HOS [16]. Interestingly, a number of Tbx5 coding sequence mutations result in relatively mild heart defects but severe forelimb abnormalities [14,17,18].

As the forelimb phenotypes of mouse *tbx5* knockouts very closely resemble the phenotype seen in moa, we reconstruct, build, and characterize moa *tbx5* to determine the molecular basis of winglessness in this extinct ratite.

## Results

### Moa *tbx5* construction and characterization

**Figure 2:**
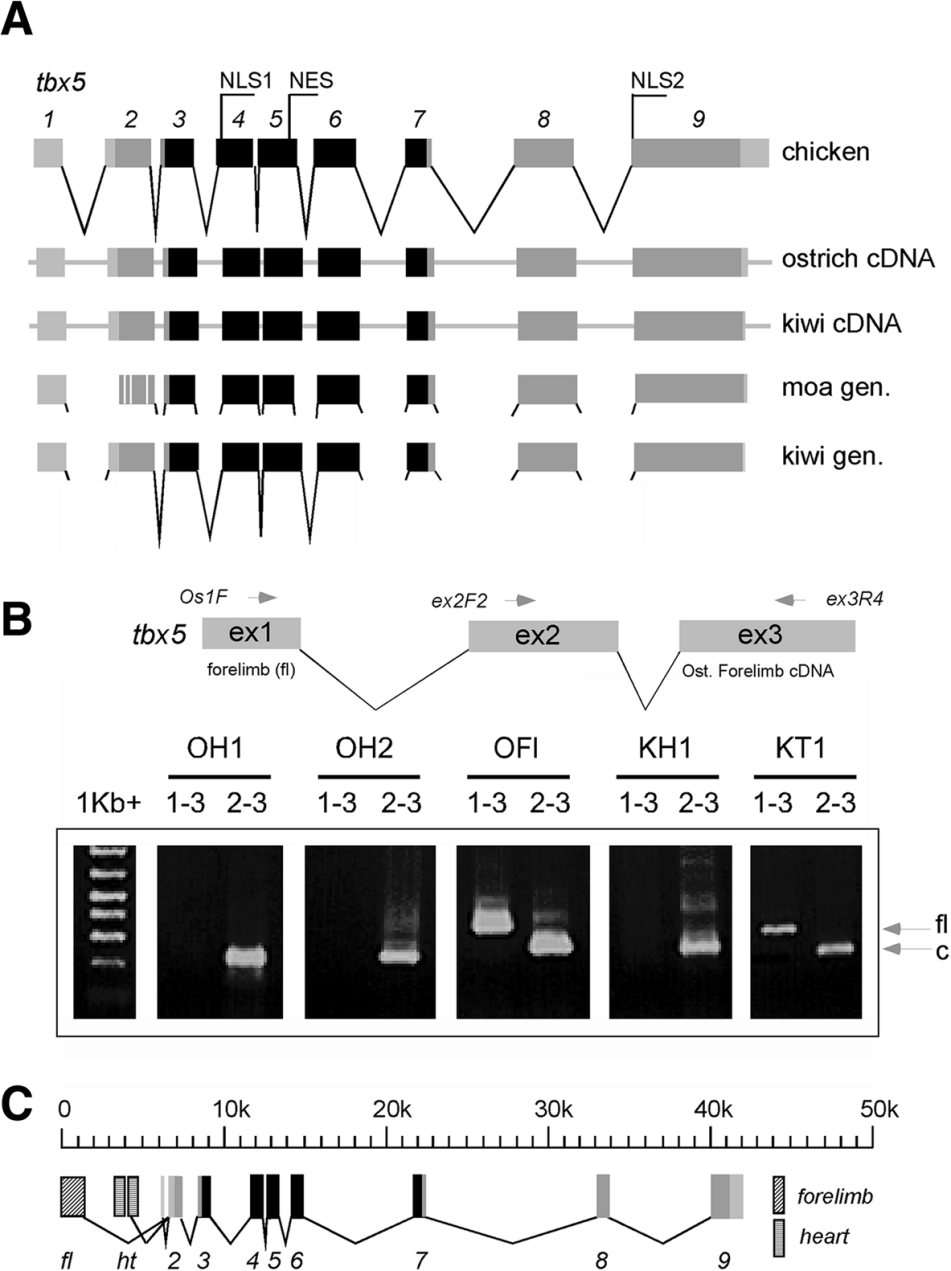
**Gene structure of moa *tbx5 *and detection of forelmb-specific *tbx5 *transcripts in ostrich and kiwi. A**. Comparison of moa *tbx5* gene structure with those of chicken, ostrich, and kiwi. Moa *tbx5* exons were identical in length to those of chicken and kiwi. Complete genome data for ostrich was not obtained. The coding region is shown in grey and is 521 amino acids long for all transcripts shown. The 183 amino acid Tbox region is shown in black and is identical between chicken, kiwi, ostrich, and moa. The four amino acids unique to moa Tbx5 at the NH_2_ terminus are shown by white vertical lines. The position of Nuclear Localisation Signals 1 and 2 (NLS1, NLS2) and the Nuclear Export Signal (NES) are shown [52,53]. **B**. A primer (OsF1) designed to *tbx5* exon1 sequences obtained from ostrich forelimb cDNA by 5’ RACE was used to try and amplify *tbx5* transcripts from heart and forelimb cDNA. Primers to exon 2 and exon 3 amplified the correct sized product from both tissues while the exon1 primer amplified transcripts from forelimb only. OH - ostrich heart cDNA, OFl - ostrich forelimb cDNA, KH - kiwi heart cDNA, KT - kiwi tissue cDNA. **C**. 5’ RACE analysis of A-tailed ostrich heart and forelimb transcripts identified forelimb and heart specific exons. Cross-hatched boxes represent exons expressed in forelimb only. Diagonally lined boxes are exons expressed in heart only. Primer walking of the chicken genome was used to determine the exon boundaries for kiwi and ostrich heart and forelimb *tbx5* cDNA. Several clones were sequenced and compared with sequences upstream of chicken *tbx5*. Two heart-specific exons were located and a single forelimb exon. Analysis of chicken sequences directly upstream of these exons detected no obvious promoter-like sequences.

To amplify moa *tbx5* sequences, primers were designed to chicken *tbx5* (NM_204173.1) and ostrich and kiwi *tbx5* mRNA. To obtain the intron/exon boundaries we first obtained these for kiwi using a range of PCR-based methods and then designed primers to these to amplify from moa (Additional file 1: Figures S1-2). Nearly all kiwi *tbx5* intron primers successfully amplified from moa aDNA. For a few intron/exon boundaries additional sequence was required from emu, cassowary, ostrich and/or rhea (Figure 2). Moa *tbx5* sequences were obtained as ~120 bp overlapping fragments from a number of species either by direct sequencing of PCR products or cloned material (Figure 2) (Additional file 1: Figures S3-5, S8). A complete sequence was obtained for the giant moa *Dinornis* with additional sequence across the highly variable regions of exon 2, exon 6, and exon 8 also being obtained from *Megalapteryx didinus* (OM Av10049). A single interspecies polymorphic position (G > C) was detected between *Dinornis* and *M. didinus* at the third base position in coding triplet 18 (Additional file 1: Figure S4). A number of additional sequence variations were detected between moa sequences, but nearly all of these were from clones and were C > T transitions commonly the result of aDNA damage. No clear heterozygosity was found amongst moa *tbx5* sequences suggesting the absence of a psuedogene (Additional file 1: Figures S4-5). Moa *tbx5* is 521 amino acids long and is identical for the essential 183 aa DNA-binding Tbox region to that of chicken, ostrich, and kiwi. Only two amino acids are unique to moa; an Aspartic acid (D) > Glutamic acid (E) change at amino acid position 3 and a Threonine (T) > Serine (S) change at amino acid position 4 (Additional file 1: Figures S6-7). To verify that these amino acid changes were present on the same molecule, ‘long’ aDNA amplications were performed to include all the variation detected in exon 2. Intron - exon boundaries of moa *tbx5* all harboured the consensus GT (donor)-AG (acceptor) splice site sequences (Additional file 1: Figure S8). A single intervening sequence change from the consensus G to A at position 5 (IVS2 + 5G > A) in *Dinornis tbx5* intron 2 has been shown to result in the human fibrinogen gamma gene (FGG) in either retention of the affected intron in the mRNA [19] or deletion of the preceding exon [20]. This sequence change however, is unlikely to have an effect on moa, as this splice site is highly conserved with that from tinamou.

### Moa Tbx5 activates *fgf10* and *ANF* promoters

**Figure 3:**
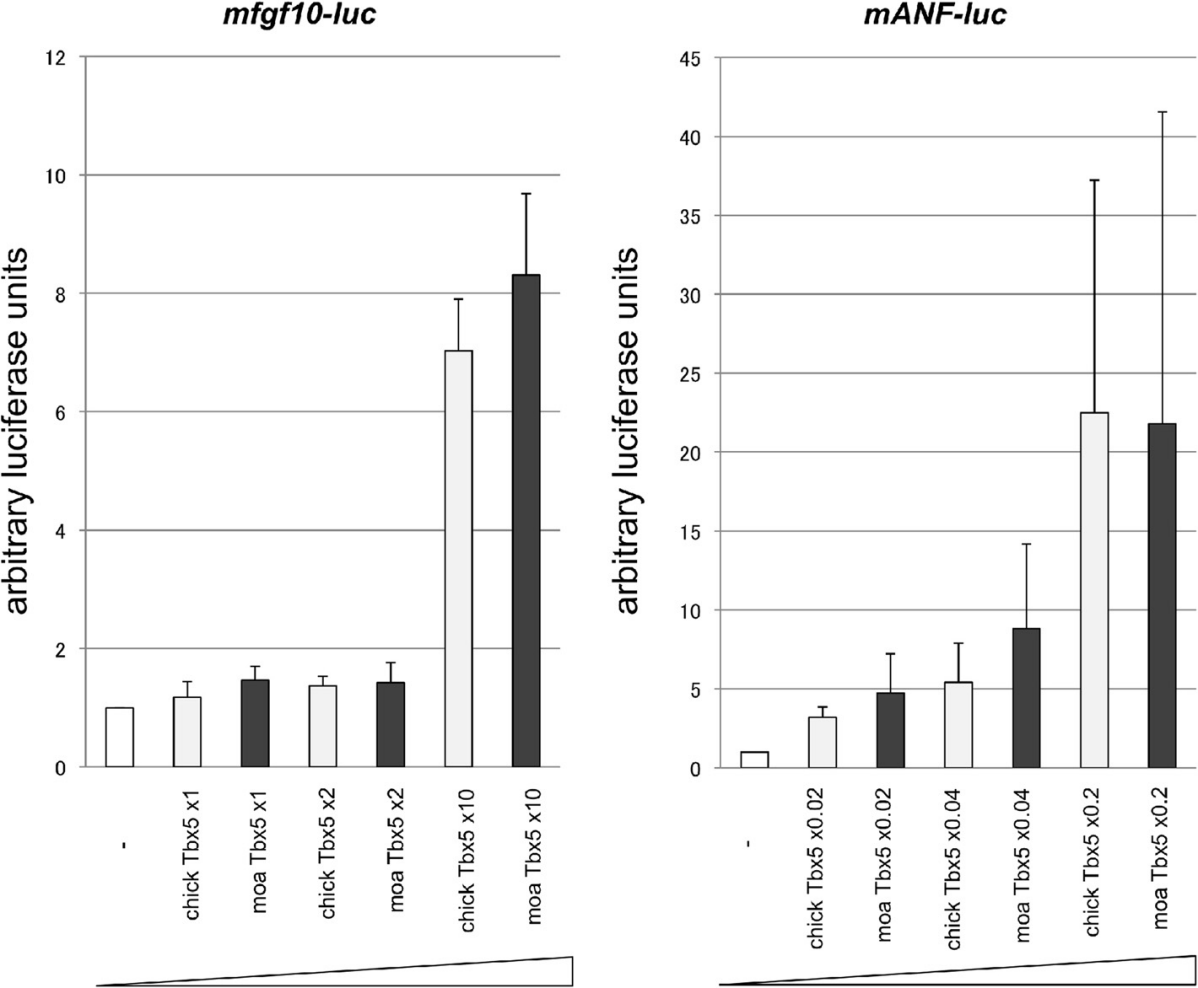
**Luciferase assay for mouse *fgf10 *and *ANF *promoters.** Both chicken and moa Tbx5 activate the *fgf10* and *ANF* promoters in a similar dose dependent manner. 1x, 2x and 10x amounts of vectors containing chicken and moa *tbx5* were transfected into HEK293T cells with *fgf10* and *ANF*-luciferase promoter constructs, and luciferase activity was measured. Both chicken and moa *tbx5* were equally effective in the activation of *fgf10* and *ANF* promoter elements.

Moa or chicken *tbx5* in pCAGGS was co-transfected into HEK293T cells with *fgf10* or *ANF* promoter-luciferase constructs and assayed for luciferase activity. Both chicken and moa Tbx5 were able to activate the *ANF* and *fgf10* promoters with equal efficiency (Figure 3).

### Moa Tbx5 induces forelimb features in chicken hindlimbs

**Figure 4:**
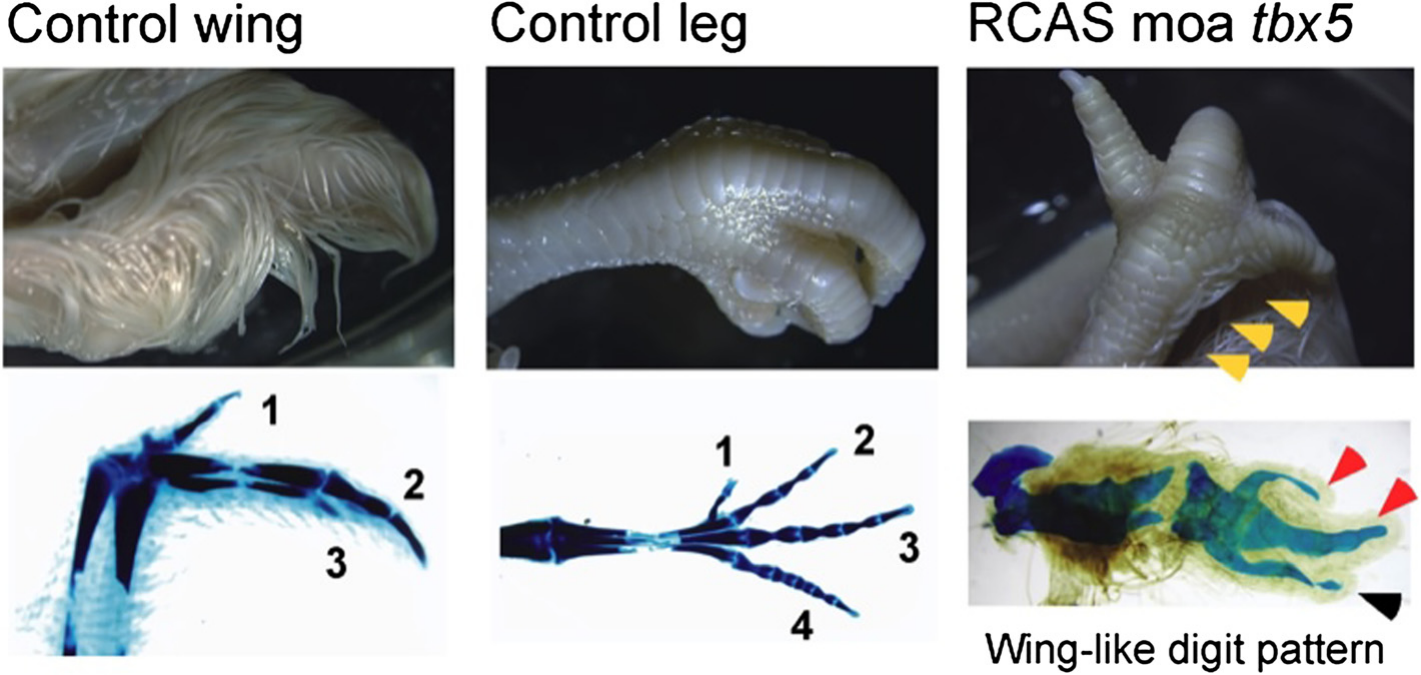
**Phenotype changes in RCAS moa *tbx5 *chick embryos.** Hamburger Hamilton (HH) stage 14 chick embryos were injected in the presumptive hindlimb region with RCAS moa tbx5 and subjected to electroporation. Embryos were harvested at HH stage 40 and stained with Victoria blue. Control HH stage 40 wing and leg are shown. Digits from Victoria blue stained control limbs are numbered. Feather growth in RCAS moa *tbx5* electroporated chick embryo hindlimbs is shown by the orange arrow heads. Victoria blue stained RCAS moa *tbx5* hindlimbs show a wing-like digit pattern with talon reduction (red arrow heads) and conversion of hindlimb digit 4 to wing-type digit 3 (black arrow head).

Electroporation of an RCAS moa *tbx5* construct into the presumptive hindlimb region of chicken embryos resulted in the hindlimb developing of a number of forelimb features, including reduced talon size, the production of feathers, and the phenotypic conversion of a hindlimb digit to one that more closely resembles a digit found in the wing (Figure 4, Table 1).

**Table 1:** **Phenotypes of chick embryo RCAS moa *tbx5 *** **at stage E16**

**Phenotype**	**Percentage (n = 9)**
Feather induction in leg	67% (6/9)
Digit 4 converted to wing-type digit 3	67% (6/9)
Talon reduction	78% (7/9)

### Determination of *tbx5* promoter activity in moa by analysis of forelimb-specific exon 1 sequences

Complementary DNAs from ostrich and kiwi heart and forelimb, respectively, were A-tailed and 5’ nested RACE was carried out to isolate *tbx5* exon 1 sequences (Additional file 1: Figure S9). Primers designed to exon 1 sequences obtained from forelimb *tbx5* only amplified from ostrich or kiwi forelimb RNA (Figure 2). Primer walking using the chicken genome allowed the isolation of ~350 bp of forelimb-specific exon 1. Isolation of this sequence from emu, cassowary, kiwi, ostrich, rhea, tinamou, and moa shows very little difference between the ratites, with only 4.7% difference shown between moa and rhea, thought to have diverged approximately 80 mya (Additional file 1: Figures S10-12; [1]).

## Discussion/Conclusions

Loss of flight is common in birds [21]. Studies on Pacific Island rails show that flightlessness can occur rapidly with some birds becoming large and flightless within three million years [22]. For ratites however, it is unknown how long it took for each species to lose flight capabilities. It has been proposed that all lost their flight independently during the Cretaceous Tertiary (KT) boundary approximately 65 mya [1], a theory that challenges the vicariance biogeography model where flightless ratites are presumed to have ‘rafted’ apart with the breakup of Gondwana [23].

Unlike the other ratites, moa have lost all elements of the forelimb skeleton, save for a finger-like bone, the scapulocoracoid, that once was part of the forelimb girdle. The scapulocoracoid consists of a short coracoid and a long tapering scapula. It is more easily identified in *Dinornis*, *Anomalopteryx*, and *Pachyornis*, but was very small due to the loss of the coracoidal fossa in *Megalapteryx*, *Euryapteryx*, and *Emeus*. Moa do not have a glenoid fossa for articulation to the humerus [24].

The timing of forelimb loss in moa is difficult to determine as a result of limited fossil material. However, by applying mutation rates to phylogenies constructed from Holocene moa, it has been suggested that the last common ancestor of Holocene moa lived 5.8-18.5 mya [25,26]. Furthermore, the discovery of moa leg bones 16–19 mya that are morphologically very similar to those from the Holocene, suggests that these moa were very similar to their later relatives and were also likely to be wingless at that time [27]. As the tinamou/moa split is thought to have occurred 60 mya [1], this places the time taken for moa to develop winglessness at approximately 40 mya. Although by no means conclusive, it is worth noting that a fossil toe bone of Late Cretaceous age (80–65 mya) from New Zealand’s North Island may possibly be from a very large bird [28].

Flightlessness and forelimb loss is thought to occur by gradual deminishment as a result of changes in expression of a number of select genes. However, high conservation between the tinamou and moa forelimb-specific exon 1 suggests that in moa *tbx5* continues to be expressed in the forelimb region despite possibly 16–19 mys of winglessness. Due to the complex nature of limb development, it has been proposed that limbs can be lost but not regained [29-31]. Collin and Cipriani (2003) [32] go on to suggest that unused genes remain in a functional state in genome for ~6 my but are almost certain to lose their function at about 10 my. This proposal has been challenged by studies on squamates where it has been shown that lost digits at least are recoverable through evolutionary time [33-35]. One reason for this may be that the pleiotropic nature of some genes may act to keep them functional [36]. For example, avian teeth were lost 70–80 million years ago, but the avian genome still harbours the genes required for tooth formation and birds are still able to do so, as has been shown by the recovery of crocodilian-like teeth in mutant *talpid*^*2*^ chickens [37]. Similarly, despite having been eyeless for over 1 million years, crosses between different eyeless cavefish populations have been shown to restore vision suggesting that even for complex structures such as eyes, very few loci are required for their redevelopment [38].

The reasons for forelimb-specific expression of *tbx5* in moa are unclear, but may suggest a role for Tbx5 in maintaining the scapulocoracoid. In flighted birds the scapulocoracoid is always separated into a coracoid and scapula that collectively function to operate flight muscles important for the wing’s downward stroke. The retention of a remnant of this structure in moa for so long may well suggest that in moa the scapulocoracoid has evolved a new function. Our results support observations made in mice showing that Tbx5 is necessary for the induction of forelimbs but not sufficient for continued forelimb development [39,40].

## Methods

### Ratite bloods, embryos, and tissues

A ~7 day post-fertilization kiwi embryo and kiwi embryonic heart were kindly made available to us by Dr. Suzanne Bassett, Otago University, New Zealand. Fertilized ostrich eggs were obtained from Kadesh Ltd, Tajo Ostrich Centre, Kumeu, Auckland, New Zealand, and incubated at 37°C with alternate clockwise/anticlockwise 180° rotations every 12 hours. Ostrich eggs were backlit to measure air-cell size reduction during embryonic growth and embryos were harvested after two weeks. (corresponding to approximate Hamburger Hamilton stage 38, (HH38; [43,44]) when most organ and limb building events are taking place). Emu, rhea, ostrich, and cassowary DNAs were a kind gift from Dr Joy Halverson, Zoogen Services, Sacramento, California, US. Kiwi blood was provided by Dr Murray Potter, Massey University, Palmerston North, New Zealand.

### Moa samples

Bone samples, kindly provided by the Otago Museum, the Auckland Institute and Museum, Canterbury Museum, and Massey University, were obtained for *D. novaezealandiae*, *D. robustus*, *E. curtus*, *E. crassus*, and *M. didinus* (see Additional file 1: Table S1).

### Ethics statement

All animal work was conducted according to relevant national and international guidelines. In particular, chick embryo work followed guidelines for animal work enforced by Nagoya University. Chicken embryos were sacrificed at HH stage 40. For the ostrich embryo work, given the early stage of sacrifice, no ethics approval was required. All waste material, including chicks, chick eggshell, and yolks were autoclaved and disposed of as industrial waste.

### Nucleic acid extraction and manipulation

Total RNA was isolated from approximately 100 mg of proximal forelimb or heart tissue using TRIzol® (Invitrogen) according to the manufacturers instructions. Genomic DNA was recovered from extant ratite blood by standard phenol/chloroform extraction and ethanol precipitation [45]. Ancient DNA (aDNA) was extracted from about 50 mg of moa bone shavings by incubation with rotation overnight at 56°C in 0.5 M EDTA/0.01% Triton X100 with ~2 mg of proteinase K. The mix was then extracted with phenol:chloroform and chloroform and the acqueous layer was purified using a Qiagen Dneasy® Blood and Tissue Kit. The silica bound aDNA was eluted with ~40 ul of 0.01% Triton X100 and stored at -20°C.

### Reverse transcription of RNA

Approximately 5 ug of total RNA was reverse transcribed at 41°C for 60 min in 20 ul volumes with random 7-mer or oligodT primers and purified by phenol:chloroform extraction and ethanol precipitation.

### Polymerase Chain Reaction (PCR)

DNA was amplified from approximately 1–20 ng of DNA as outlined in [46]. A number of PCR-based methods were used to obtain the kiwi *tbx5* intron/exon boundaries required to allow primer design for moa amplification. These included: *Single primer PCR* - A single primer was used in a standard PCR reaction at low annealing temperatures for one cycle to allow random priming and then amplified for ~35 cycles with the annealing temperature set at the primer’s T_m_. *Hairpin primer ligation PCR* - hairpin primers containg a *Pst*I compatible overhang were ligated to *Pst*I-digested DNA before standard PCRs were carried out using the hairpin primer and a *tbx5*-specific primer. *Medium range PCR* - amplification across the smaller *tbx5* introns was carried out usng Elongase® (Invitrogen) or Expand Long Template PCR System (Roche) as outlined by the manufacturer. *Inverse PCR* - Inverse PCR was carried out to obtain intron sequences directly from moa. Biefly, moa aDNA was denatured, then dephosphorylated with shrimp alkaline phosphatase (SAP) before fresh phosphates were added with T4 Polynucleotide Kinase. The aDNA was then circularised with Circligase^TM^ ssDNA Ligase (Epicentre®) and subjected to rolling circle amplification using Templify^TM^ (Amersham). The amplified aDNA was then subjected to standard PCR using inverse primers.

### Construction strategy for moa *tbx5*

To isolate moa *tbx5* sequences we initially obtained full-length *tbx5* cDNA sequences for kiwi and ostrich. Comparison with the chicken genome identified the intron/exon boundaries and primers were then designed to recover moa *tbx5* exon sequences. To obtain moa *tbx5* intron/exon boundaries, these boundaries were first isolated from kiwi. Primers were then designed to kiwi *tbx5* intron sequences and conserved ostrich/kiwi/chicken *tbx5* exon sequences to obtain moa *tbx5* intron/exon boundary sequences. Most primers designed to kiwi *tbx5* intron sequences successfully amplified from moa aDNA. For a few introns however, primers successful for amplification from moa required additional intron sequences from rhea, and/or ostrich.

### Nested 5’ RACE

Total RNA isolated from embryonic ostrich and kiwi forelimb and kiwi heart were A-tailed with terminal transferase (Invitrogen) and amplified with H5FdT (5’- AATCGGACAAACTGGTCCTTGCAACdT_20_) and ex2R2 (5’- GGTGAGCGACTTGCTGGTG), followed by H5F (5’- AATCGGACAAACTGGTCCTTGCAAC) and ex2R3 (5’- CAAAGCCTTCCTCCGTAT). Amplified products were TA cloned into pGEM®T-Easy (Promega) and sequenced with m13F (5’-TGTAAAACGACGGCCAGT) or m13R (5’-CAGGAAACAGCTATGACC). Full-length forelimb-specific exon 1 transcripts were then obtained by cDNA walking using primers designed to conserved upstream regions of the chicken genome.

### Sequencing

PCR products were purified by centrifugation through dry Sephacryl S200HR, sequenced using ABI BigDye® Terminator v3.1 chemistry, then analysed and aligned in Sequencher^TM^ 5.0 (Gene Codes Corporation).

### Ancient DNA procedures

In accordance with criteria suggested for the verification of aDNA sequences [47], a number of samples were extracted and sequenced at a separate ancient DNA facility at Massey University, Auckland, New Zealand.

### Activation of *fgf10* and *ANF* promoters by moa Tbx5

The ability of moa Tbx5 to bind to and activate downstream *fgf10* and *ANF* promoters was determined using a luciferase assay. HEK293T cells were seeded into 24-well plates at a density of 5 × 10^4^ cells/well twenty four hours before transfection. The following vectors were transfected into HEK293T cells using XtremeGENE HP (Roche); 6 kb of mouse sequence immediately upstream of *fgf10*[6], or 0.7 kb of mouse sequence immediately upstream of the *ANF* promoter [7], both fused to luciferase, pCAGGS containing chick or moa *tbx5*, CMX-*β-*galactosidase, and an empty control vector (pcDNA3.1). Forty-eight hours after transfection, the cells were lysed, and luciferase activity was measured using a Luminescencer-JNR (ATTO). β-galactosidase activities were measured to standardize the efficiency of transfection. Results are expressed as the average of three samples including standard deviation.

### Miss-expression of moa *tbx5* in chicken

Electroporation into the chick hindlimb field was carried out as described previously [48]. Briefly, 2 ug/ml of RCAS-moa *tbx5* plasmid was injected into the prospective hindlimb field at Hamburger Hamilton (HH) stage 14 by glass capillary. Electric pulses (8 V, 60 ms pulse-on, 50 ms pulse-off, three repetitions) were applied using an CUY21-EDIT electroporator (NAPA GENE) with platinum electrodes. Electroporated embryos were harvested at HH stage 40 and stained with Victoria blue. Victoria blue staining was carried out as described in [49].

### Availability of supporting data

Supporting tables and figure are available as additional files. *Tbx5* sequences are deposited in NCBI’s GenBank [GenBank Acc Nos. KJ584152-KJ584161].

## Competing interests

The authors declare that they have no competing interests.

## Authors’ contributions

LH, TS, TO, YW, and SM carried out the molecular genetic studies. LH, DML, CDM, CS, and MH helped conceive the study and participated in its design and coordination, and helped to draft the manuscript. All authors read and approved the final manuscript.

## Supplementary Material

Additional file 1 **Moa *tbx5; *** **materials, methods and sequences.** Detailed descriptions of materials and methods used, nucleotide and amino acid sequences. Click here for file
