# Supplementary material for: Reconstruction and in vivo analysis of the extinct tbx5 gene from ancient wingless moa (Aves: Dinornithiformes)
Source: BMC Evol Biol. 2014 May 14;14:75. doi: 10.1186/1471-2148-14-75 (PMC4101845; doi:10.1186/1471-2148-14-75)
Supplement: Additional file 1 — Moa tbx5; materials, methods and sequences. Detailed descriptions of materials and methods used, nucleotide and amino acid sequences. [file 1471-2148-14-75-S1.docx]

**Additional file**

**Reconstruction and *in vivo* analysis of the extinct *tbx5* gene from ancient wingless moa (Aves: Dinornithiformes)**

**Leon Huynen^1^, Takayuki Suzuki^2^, Toshihiko Ogura^3^, Yusuke Watanabe^3^, Craig D Millar^4^, ^5^Michael Hofreiter, Craig Smith^6^, Sara Mirmoeini^7^ and David M Lambert^1^***

[^1^L.huynen@griffith.edu.au](mailto:1L.huynen@griffith.edu.au) [D.lambert@griffith.edu.au](mailto:D.lambert@griffith.edu.au) Environmental Futures Centre, Griffith University, 170 Kessels Road, Nathan, Qld 4111 Australia. ^2^[suzuki.takayuki@j.mbox.nagoya-u.ac.jp](mailto:suzuki.takayuki@j.mbox.nagoya-u.ac.jp) Division of Biological Science, Nagoya University, Nagoya, Japan 464-8602. [^3^Ogura@idac.tohoku.ac.jp](mailto:3Ogura@idac.tohoku.ac.jp) [ywatanabe@idac.tohoku.ac.jp](mailto:ywatanabe@idac.tohoku.ac.jp) Institute of Development, Aging and Cancer (IDAC), Tohoku University, Sendai 980-8575, Japan. [^4^CD.Millar@auckland.ac.nz](mailto:4CD.Millar@auckland.ac.nz) Allan Wilson Centre for Molecular Ecology and Evolution, School of Biological Sciences, University of Auckland, Private Bag 92019, Auckland, New Zealand. [^5^michi@palaeo.eu](mailto:5michi@palaeo.eu) Department of Biology, University of York, YO10 5DD, UK and Faculty of Natural Sciences, University of Potsdam, 14476 Potsdam, Germany. [^6^craig.smith@mcri.edu.au](mailto:6craig.smith@mcri.edu.au) Murdoch Children’s Research Institute, Royal Children’s Hospital, Flemington rd Parkville, Victoria 3052, Australia. [^7^saramoeini@hotmail.com](mailto:7saramoeini@hotmail.com) Institute of Natural Sciences, Massey University, Auckland 0632, New Zealand.

* corresponding author

**Materials and Methods**

**Materials**

**Ratite bloods, embryos, and tissues.** Red-blood cell enriched kiwi bloods were a kind gift from Dr Murray Potter, Massey University, Palmerston North, New Zealand. Fertilized ostrich eggs were obtained from Kadesh Ltd, Tajo Ostrich Centre, Kumeu, Auckland, New Zealand and incubated at 37°C for two weeks. The eggs were rotated clockwise, then anticlockwise 180° every 12 hrs to prevent toxin buildup within the egg. The egg was opened using a dremel and the embryo sacrificed by decapitation. Tissue from the heart and forelimb was removed by scalpel and total RNA was isolated from approximately 100mg of each tissue using TRIzol® (Life Technologies). A number of kiwi embryos and a preserved embryonic kiwi heart were kindly made available to us by Dr. Suzanne Bassett (Otago University, New Zealand). One kiwi embryo (K54-38) proved to be a good source of RNA (as judged by the yield of full-length rRNA by standard agarose gel electrophoresis). The structural features of this kiwi embryo were difficult to identify however, so a series of small samples were removed from several equidistant areas on the outside of the embryo and then pooled for RNA extraction.

**Ratite DNAs**. Emu, cassowary, ostrich, and rhea DNAs were kindly provided by Dr Joy Halverson, Zoogen, Sacramento, California, US. Tinamou major DNA samples (225 EDA, 106 11-12-10) were gratefully received from Prof. Siwo de Kloet, Dept of Biological Science, Florida State University, Tallahassee, US.

**Table S1**. **Moa samples used to sequence *tbx5*.** Previous work had shown that the moa samples shown below provided high yields of good quality nuclear DNA (Huynen et al, 2003). Samples were originally sourced from Canterbury Museum (CM), the Auckland Institute and Museum (AIM), and Massey University (MU).

| **Museum ID #** | ***Species*** | **Bone** | **Notes** |
| --- | --- | --- | --- |
| CM Av8317 | *Emeus crassus* | femur | Pyramid Valley, SI |
| CM Av8378 | *Euryapteryx curtus* | femur | Pyramid Valley, SI |
| OM Av10049 | *Megalapteryx didinus* | femur | Serpentine Range, SI, 1608±40 yrBP |
| CM Av9032 | *Dinornis robustus* | femur | Oamaru, SI |
| CM Av30495 | *Dinornis robustus* | femur | Waikari, SI, juvenile |
| CM Av30875 | *Dinornis robustus* | femur | Glen Mae, SI, juvenile? |
| AIM B6316 | *Dinornis novaezealandiae* | femur | Waikaremoana, NI |
| AIM B7037 | *Dinornis novaezealandiae* | femur | Puketiti, NI |
| AIM B7070 | *Dinornis novaezealandiae* | femur | Doubtless Bay, NI |
| AIM B7072 | *Dinornis novaezealandiae* | femur | Kawhia, NI |
| AIM B7145 | *Dinornis novaezealandiae* | femur | Waitomo, NI |
| CM Av17563 | *Dinornis novaezealandiae* | femur | Makara, NI, subadult? |
| MU DnTbT | *Dinornis novaezealandiae* | tibiotarsus | Opiki, NI |

**Methods**

**Nucleic acid extraction**. DNA was extracted from ratite blood using standard SET / proteinase K, phenol:chloroform methods as outlined in Sambrook and Russell (2001). Total RNA was extracted from ostrich and kiwi tissue with TRIzol® (Invitrogen) according to the manufacturers instructions. Ancient DNA was extracted in a physically isolated and purpose-built Ancient DNA Laboratory at Griffith University, Queensland. Approximately 50 mg of bone was shaved from the bone surface and incubated with rotation overnight at 56°C in 0.4 ml of 0.5 M EDTA / 0.01% Triton X100, and ~2 mg of proteinase K. The mix was then extracted with phenol:chloroform and chloroform and then purified by silica bed binding using a Qiagen Dneasy® Blood & Tissue Kit. The aDNA was eluted from the column with ~40 ul of 0.01% Triton X100 and stored at -20°C.

**Reverse transcription of RNA**. Approximately 5ug of total RNA was reverse transcribed into cDNA in a 20 ul volume containing 200 ng of random 7mer primers (or oligodT), 400 uM of each dNTP, 50 mM Tris-Cl pH 8.3, 75 mM KCl, 3 mM MgCl_2_, 5 mM DTT, 100 ug/ml BSA, and 200 U of MMLV reverse transcriptase. The mix was incubated at 41°C for 1 hour and then purified by phenol:chloroform extraction and ammonium acetate / ethanol precipitation, and resuspended in 25 ul of MQ H_2_O.

**cDNA tailing**. cDNAs (approximately 5 ul of the reverse transcription reaction, above) were tailed with 200 uM dATP and 5 U of recombinant terminal deoxynucleotidyl transferase (rTdT; Invitrogen) in 20 ul volumes containing 100 mM potassium cacodylate, 2 mM CoCl_2_, and 0.2 mM DTT pH 7.2. The mix was incubated at 37°C for one hour and then purified by phenol:chloroform extraction and ethanol precipitation.

**Polymerase Chain Reaction (PCR)**. Unless stated otherwise all PCR amplifications were carried out in 10-20 ul reactions containing 50 mM Tris-Cl pH 8.8, 20 mM (NH_4_)_2_SO_4_, 2.5 mM MgCl_2_, 1 mg/ml BSA, <20 ng of template DNA, 200 uM of each dNTP, 0.5 uM of each primer, and 0.3 U of Platinum Taq polymerase (Invitrogen). Where greater specificity was required Betaine and / or DMSO were added to 1 M and 5% respectively. Semi-nested PCRs (used to check identity or purify PCR products) were carried out by adding ~1 ul of the initial PCR mix to a fresh PCR mix containing one of the original primers and an internal primer. The fresh PCR mix was amplified for 10 - 15 cycles. All amplification reactions were carried out in thin-walled tubes in an ABI GeneAmp® PCR System 9700, and PCR products were usually separated by electrophoresis in 1% std / 1% LMP agarose in 0.5 x TBE, then stained with 50 ng/ml ethidium bromide and visualised over UV light. To obtain *tbx5* intron / exon boundaries for primer design for amplification from moa, various PCR-based methods were used on ratite genomic DNA (Figure and below).

**
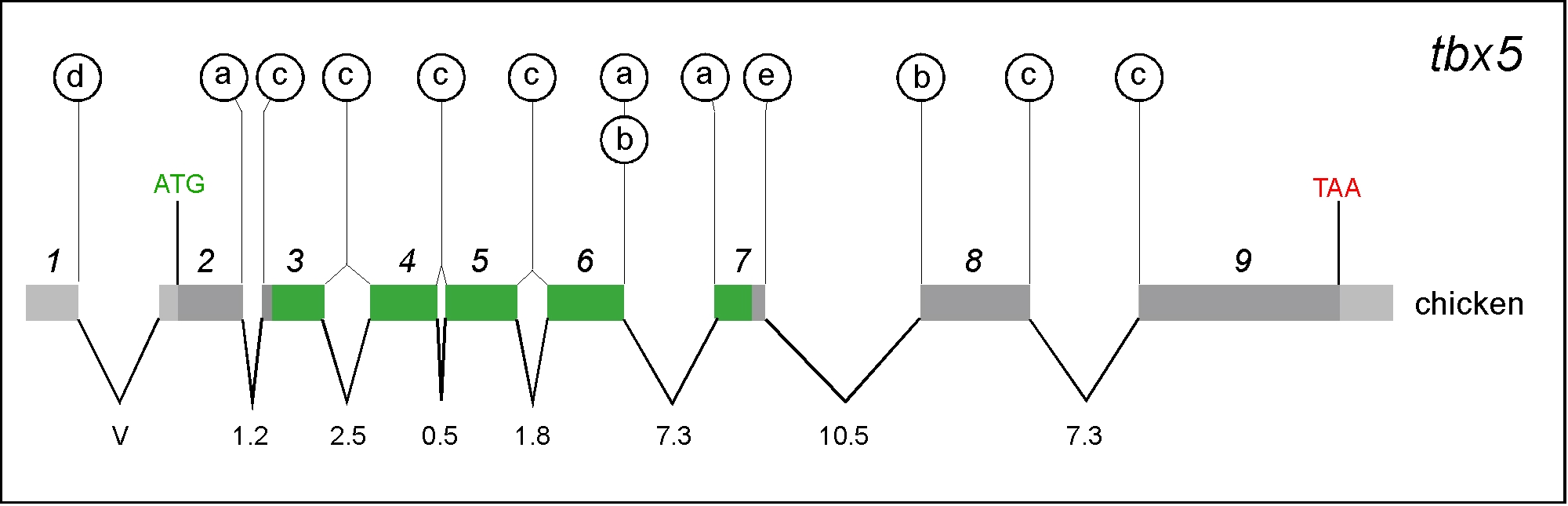
**

**Figure S1**. **Construction strategy for moa *tbx5***. The strategy for obtaining the coding sequence for moa *tbx5* consisted of obtaining *tbx5* coding and (where required) *tbx5* intron sequences from the closely related ratites kiwi, rhea, emu, ostrich, and cassowary. Most primers used to obtain the moa *tbx5* intron / exon boundaries were designed from kiwi sequences (Figure S3). To obtain the kiwi *tbx5* intron sequences a number of PCR-based methods were used (labeled in circles). These included; ^a^Single primer PCR, ^b^Hairpin primer ligation, ^c^Medium range PCR, ^d^dC PCR, and ^e^Inverse PCR (see below). Where required amplification products were isolated from agarose and cloned for sequencing. Exons are numbered and shown as boxes (not to scale). Intron size (kb) is shown at the bottom. The size of intron one is dependent on the exon used and is shown as V (variable). Start (ATG) and stop (TAA) codons are marked. Light grey areas represent the 5’ and 3’ untranslated regions. Green represents the DNA-binding T-box region.

**^a^Single primer PCR.** Three kiwi intron / exon boundaries were obtained using simple single primer PCR. Separate PCR mixes containing 5 mM MgCl_2_, ~0.3 U Vent_R_® (exo-) DNA Polymerase (NEB), and either ex2F (5’- GATTCGGCGAAGGAAGCTCGT), ex6F (5’- CTCCATGCACAAATACCAGCC), or ex7R (5’- TGCATCCTGGACATCCTGTG) were denatured at 94°C for 2 min and the primers were allowed to anneal at 30°C for 5 min and then extend for 2 min at 72°C. 1 volume of water was then added to the mix and the reaction was subjected to 35 cycles of; 94°C 20 sec, 60°C 20 sec, 72°C 20 sec. A second (nested) PCR was then carried out using the original primer and an internal primer ex2F4 (5’- AAAGAGCTGCAGGCTGAAA), ex6F3 (5’- CTCCACATCGTGAAAGCGGACGAGAA), or ex7R2 (5’- TGTGGAGCTCCATGTCGTC) respectively.

**^b^Hairpin primer ligation.** For two intron / exon boundaries hairpin primer ligation and PCR was carried out. Kiwi DNA was partially digested with *Pst*I and then ligated to the hairpin primer *Pst*I-hp2 (5’- GCTCGATCCTAGGATCGAGCTGCA). *Pst*I was chosen to give fragments in the range of 1.0 - 2.0 kb. The ligated fragments were then subjected to PCR with *Pst*I-hp2 and one of the exon-specific primers ex6F4 (5’- TGCACCCACGTCTTCC) or ex8R3 (5’- CCTGGTCTCACCACTGAATG).

**^c^Medium range PCR.** Introns that ranged in size from 0.5 kb to 7.3 kb were directly amplified using primers designed to the flanking exons. Primer pairs ex2F3 (5’- ATGCCGAGGAAGGCTTT) / ex3R (5’- CAGCCTTTGTTATGATCATCT), intron 2; ex3F4 (5’- AAAAGTGTTTTTGCACGAGCG) / ex4R4 (5’- TCATCCGCTGGTACAATATCCA), intron 3; ex4lrF (5’- GATATTGTACCAGCGGATGACC) / ex5lrR (5’- GAAACCAGCTGCCTCATCC), intron 4; ex4F (5’- CCCAGTTACAAAGTGAAGGT) / ex5R (5’- GGTGAGCTTGAGCTTCTGGAA), intron 4; ex5lrF (5’- ACTGGATGAGGCAGCTGGTTTCC) / ex6R4 (5’- AGCGATGAAGGCAGTCTCGGG), intron 5; and ex8F (5’- GTTGTTCCCAGGAGCACAGTGA) / ex9R3 (5’- AGTCCTGTATGAAGTGTTCAGTCC); intron 8 were used to amplify complete introns in 20 ul reactions containing with either Platinum Taq (Invitrogen), Expand Long Template System (Roche) or Elongase® (Invitrogen).

**^d^dC PCR.** To obtain the intron / exon boundary for exon 1, we used a cytosine rich primer AnchdC (5’-GCTCGATCCTAGGATCGAGC_12_) to encourage binding to the GC rich areas common to 5’ intron boundaries and oex1F2 (5’- TCGGTTTATTTGCATCGTT).

**^e^Inverse PCR.** Inverse PCR was used to obtain the flanking sequence of exon 7 which was difficult to obtain by other methods. In the process we developed a method for making large amounts of aDNA. In general, approximately 100 mg of bone shavings will provide about 100 ng of ancient DNA, a large proportion of which will be contaminating microbial DNA. This provides enough DNA for approximately 50 – 100 PCR reactions. As this work required the testing of numerous primers and the optimisation of a number of methods, a large amount of aDNA would be benificial. For this reason we tried to generate large amounts of aDNA by circularization of the aDNA and rolling circle amplification. In this was we were able to produce micrograms of aDNA from nanograms of starting material. The technique relies on the denaturation of ancient DNA and then the removal of terminal phosphates, a significant proportion of which will be damaged. Fresh phosphates are then added and the single stranded DNA (ssDNA) is subjected to intra-specific ligation using the ssDNA ligase CircLigase. Circular molecules are then amplified using random primers and the highly processive polymerase phi29. In this way we have achieved at least 1000 fold increases in whole genome aDNA. An added advantage of this method is that it allows the direct determination of unknown flanking sequences by inverse PCR (iPCR). Furthermore PCR of the amplified aDNA typically results in the production of DNA concatemers, which have proved useful for sequencing, as sequence is obtained directly adjacent to the sequencing primer.

**
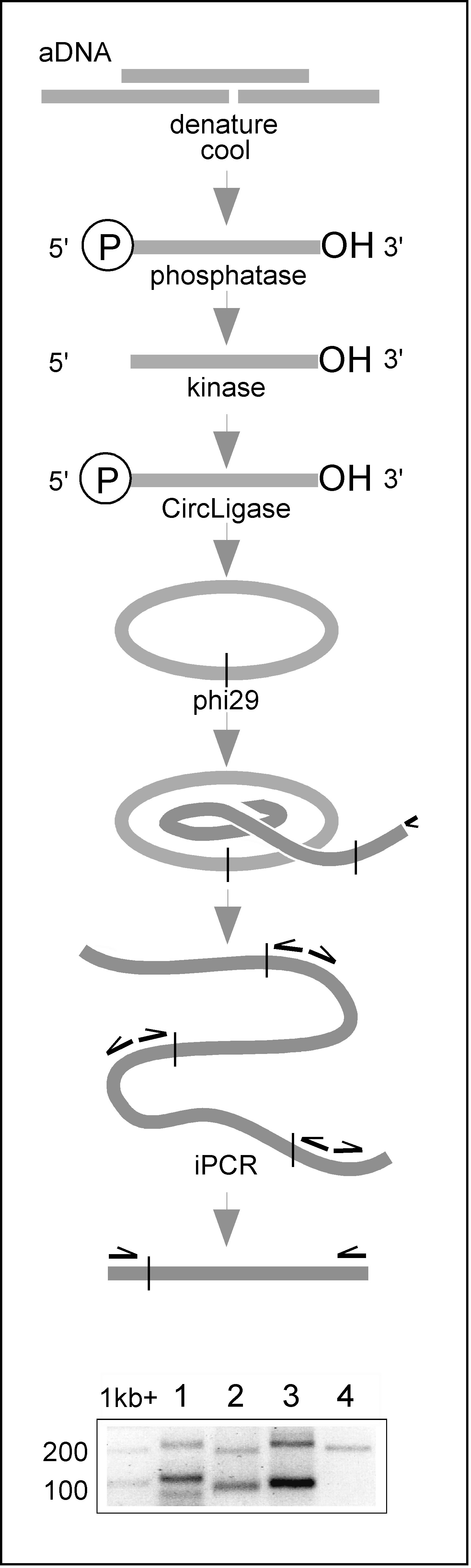
Figure S2. Amplification and inverse PCR of aDNA**. **Top** Approximately 5 ul (5 ng) of ancient DNA was denatured in 10 ul of Circligase buffer at 94°C for 1 min and then cooled on ice. The aDNA was dephosphorylated by incubation with ~1 U of shrimp alkaline phosphatase (SAP; ) at 37°C for 15 min and the SAP was inactivated by incubation at 65°C for 5 min. Fresh phosphates were then added by incubating at 37°C for 15 min with 2 U T4 polynucleotide kinase and 200 uM ATP. The ssDNA was subsequently circularized by incubation at 60°C for 1 hour with 100 U CircLigase^TM^ single-stranded DNA ligase (Epicentre®), and 2 ul of the mix was amplified overnight at room temperature using random primers and phi29 polymerase as provided by the Templify^TM^ kit (Amersham). We typically obtained a few micrograms of amplified aDNA from about 5 ng of starting material. Moa specific targets were then amplified by inverse PCR. **Bottom**. Approximately 5 ng of amplified aDNA from AIM B6316 or CM Av30495 was subjected to inverse PCR using *tbx5* exon 7 primers ex7Rrev (5’-CACAGGATGTCCAGGAT) and ex7R3 (5’- CGTCACTGCCGCGGAAACCTT) or ex7F4 (5’-CAGTGACGACATGGAGCT) and ex7R3 (lanes 1 and 2). Lanes 3 and 4 are control amplifications of moa mitochondrial DNA.

**Cloning**. PCR products were routinely cloned into the vectors pGEM®-T Easy (Promega), pUC19, pCR®2.1(TA) or pCR®2.1-TOPO (Invitrogen) using chemically competent DH5a, SURE® (Stratagene) or One Shot® Mach1^TM^ T1 cells (Invitrogen) and plated onto Ampicillin plates (100 ug/ml). Positive colonies were selected by colony PCR using the primers M13F (5’- TGTAAAACGACGGCCAGT) and M13R (5’- CAGGAAACAGCTATGACC).

**Sequencing**. PCR products were purified by passage through dry Sephacryl S200HR, sequenced using ABI BigDye® Terminator v3.1 chemistry, then analysed and aligned in Sequencher^TM^ 5.0 (Gene Codes Corporation).

**Ancient DNA procedures**. In accordance with criteria suggested for the verification of aDNA sequences (Cooper and Poinar, 2000), a number of samples were extracted and sequenced at a separate ancient DNA facility at Massey University, Auckland, New Zealand.

**Transfection of chick hindlimbs with moa *tbx5*.** Electroporation into the chick hindlimb field was carried out as described in Suzuki and Ogura (2008). Approximately 2 ug/ml of purified RCAS-moa *tbx5* plasmid was injected into the prospective hindlimb field at Hamburger Hamilton (HH) stage 14 by glass capillary. Electric pulses (8 V, 60 ms pulse-on, 50 ms pulse-off, three repetitions) were applied using an CUY21-EDIT electroporator (NAPA GENE) with platinum electrodes. Electroporated embryos were harvested at HH stage 40 and stained with Victoria blue. Victoria blue staining was carried out as described in Suzuki et al (2008).

**Figure S3**. ***tbx5* coding sequences from ostrich and kiwi mRNA**. Approximately 5ug of total RNA was reverse transcribed into cDNA and amplified with the primers shown. Dashes - identical sequence to the chicken reference cDNA (GenBank acc. no. NM_204173), Blue - forward primers, red - reverse primers. In most instances primers designed to chicken *tbx5* worked well with both kiwi and ostrich. However, in some cases, specific primers were required (eg ex8R2 and ex9R4). The start codon (ATG) is shown in green and the stop codon (TAA) in red. Approximate position (🡻) and size (kb) of the introns was determined by comparison of the chicken *tbx5* mRNA with the chicken genome (Build 3.1). Unreadable sequence at the 3’ terminus is shown by a ‘?’. ck hrt - chicken heart, os hrt - ostrich heart, os fl - ostrich forelimb, ki fl - kiwi forelimb (K54-38). *Tbx5* sequences from ostrich heart and forelimb were identical.

ex2F>

ck hrt 1 GGGGGATTCGGCGAAGGAAGCTCGTAAC**ATG**GCGGACACCGAGGAAGGCTTCGGGCTCCCGAGCACGCCGGTTGACTCGGAGGCCAA

os hrt 1 --------------------------------T--G-----------T--------A-C--------C------C---T----

os fl 1 --------------------------------T--G-----------T--------A-C--------C------C---T----

ki fl 1 --------------------------------T--------------T----------C--------C----------T----

ck hrt 88 GGAGCTGCAGGCTGAGGCCAAGCAGGATCCCCAGCTGGGGACCACCAGCAAGGCCCCCACCTCTCCACAGGCGGCCTTCACCCAGCA

os hrt 88 A--------------AA----------CA-T--A------G-----------T-G-----------C-----A--------------

os fl 88 A--------------AA----------CA-T---------G-----------T-G-----------C-----A--------------

ki fl 88 A--------------AAG---------CA-T--A------G-----------T-------------C--------------------

🡻 1.2 kb ex3F3>

ck hrt 175 GGGCATGGAGGGGATCAAAGTGTTTTTGCACGAGCGGGAGCTGTGGCTGAAATTTCACGAGGTGGGGACGGAGATGATCATAACAAA

os hrt 175 ---------------A------------------------T-------------------A--------T--------T--------

os fl 175 ---------------A------------------------T-------------------A--------T--------T--------

ki fl 175 ------------C--A--------------------------------------------A--------C-----------------

🡻 2.5 kb <ex4R

ck hrt 262 GGCTGGAAGGCGTATGTTTCCCAGTTACAAAGTGAAGGTCACTGGACTCAATCCAAAAACGAAGTACATACTGTTGATGGATATTGT

os hrt 262 ------------------C-----------------------------T-----------T--------------------------

os fl 262 ------------------C-----------------------------T-----------T--------------------------

ki fl 262 ------------------C-----------------------------T-----------T--------------------------

🡻 0.5 kb ex5F>

ck hrt 349 ACCAGCGGATGACCACAGATACAAATTTGCAGATAATAAATGGTCCGTGACCGGGAAGGCAGAACCGGCCATGCCCGGCCGCCTCTA

os hrt 349 ---------------------------------------------G-----A-----------G-----------------GT-G--

os fl 349 ---------------------------------------------G-----A-----------G-----------------GT-G--

ki fl 349 ---------------------------------------------G-----A-----------G-----------------G--G--

<kx5lrR

ck hrt 436 CGTGCACCCCGACTCCCCCGCTACTGGAGCCCACTGGATGAGGCAGTTGGTTTCCTTCCAGAAGCTCAAGCTCACCAACAACCACCT

os hrt 436 ---C-----------------C--C--C------------------C----------T--A--A-----------------------

os fl 436 ---C-----------------C--C--C------------------C----------Y--A--A-----------------------

ki fl 436 ---C-----------------C--C--C------------------C-------------A--A-----------------------

🡻 1.8 kb ex6F>

ck hrt 523 TGACCCCTTCGGACATATCATCCTGAACTCCATGCACAAATACCAGCCCCGGCTCCACATCGTGAAGGCGGATGAGAACAACGGCTT

Os hrt 523 C-----------------------------------------------------------------A--A--C--------------

Os fl 523 C-----------------------------------------------------------------A--A--C--------------

ki fl 523 C-----------------------------------------------------------------A-----C--------------

<ex6R5 <ex6R 7.3 kb 🡻

ck hrt 610 TGGCTCCAAGAACACTGCCTTCTGCACCCATGTCTTCCCCGAGACTGCCTTCATCGCTGTTACCTCCTACCAAAACCACAAGATCAC

os hrt 610 C--G-----------C-----T--------C--------G-----C-----------C--C--------------------------

os fl 610 C--G-----------C-----T--------C--------G-----C-----------C--C--------------------------

ki fl 610 C--G-----------C-----T--------C--------G-----C-----------C--C--------------------------

ck hrt 697 TCAGCTGAAGATTGAGAACAACCCCTTCGCAAAAGGTTTCCGCGGCAGCGATGACATGGAGCTCCACAGGATGTCCAGGATGCAGAG

os hrt 697 C---T-A--------------------T--G-----------------T--------------------------------------

os fl 697 C---T-A--------------------T--G-----------------T--------------------------------------

ki fl 697 C---T-A--------------------T--G-----------------T--C-----------------------------------

🡻 10.5 kb ex8F> <ex8R3

ck hrt 784 TAAAGAGTACCCAGTTGTTCCCAGGAGCACAGTGAGACAGAAAGTGTCCTCGAATCACAGCCCCTTCAGCGGTGAGACCAGGGTCCT

os hrt 784 ------------G--------------------------A-----------A-----------G-----------------------

os fl 784 ------------G--------------------------A-----------A-----------G-----------------------

ki fl 784 ------------G--------------------------A-----------A-----------A-----T-----------------

<ex8R2

ck hrt 871 TTCCACCTCCTCCAACCTGGGCTCCCAGTACCAGTGTGAGAACGGGGTGTCAAGCACCTCCCAGGACCTGCTGCCGCCCACCAACCC

os hrt 871 ----G-----------T----G--------T--A--C--------------G--T---------------T-A-----TG-------

os fl 871 ----G-----------T----G--------T--A--C--------------G--T---------------T-A-----TG-------

ki fl 871 ---TG-----------T----G-----R--T--R--C--------------G-----Y-------R----Y-R-----TG-------

🡻 7.3 kb ex9F>

ck hrt 958 CTACCCGATCTCCCAGGAGCACAGCCAGATCTACCACTGCACCAAGAGAAAAGATGAGGAATGTTCCACCACCGAGCATGCCTACAA

os hrt 958 G-----------------------------------------------------------G--------------------------

os fl 958 G-----------------------------------------------------------G--------------------------

ki fl 958 G-----------S------------------------------------------A-------------------------------

<ex9R3

ck hrt 1045 GAAGCCCTACATGGAAACTTCACCAGCGGAAGAGGATCCTTTCTACAGGTCCAGTTACCCCCAGCAACAGGGACTGAACACTTCGTA

Os hrt 1045 ---------------------T--G--A--------------------------------------G-----------------A--

Os fl 1045 ---------------------T--G--A--------------------------------------G-----------------A--

ki fl 1045 ---------------------T--G--A--------------------------------------G-----------------A--

ck hrt 1132 CAGGACTGAATCAGCCCAGCGCCAGGCATGTATGTACGCCAGCTCTGCTCCCCCCACGGACCCCGTGCCCAGCCTGGAAGACATCAG

Os hrt 1132 ---------------T--------A--------------------G-----------------------------A-----------

Os fl 1132 ---------------T--------A--------------------G-----------------------------A-----------

ki fl 1132 ---------------T--------A--------------------G-----------------------------A-----------

ck hrt 1219 CTGTAACACGTGGCCCAGCGTGCCGTCCTACAGCAGTTGCACAGTGTCTGCCATGCAGCCCATGGACAGGTTACCCTACCAGCATTT

Os hrt 1219 ---------------G--------C-----------------------------------G--------------------------

Os fl 1219 ---------------G--------C-----------------------------------G--------------------------

ki fl 1219 ---------------G--------C--------------------A--------------G--------------------------

ck hrt 1306 CTCTGCCCACTTCACCTCGGGGCCTCTGATGCCCCGGCTCAGCAGCGTGGCCAACCACACGTCCCCCCAGATAGGAGACACCCATAG

Os hrt 1306 ------------------T-----G-----------T--------------------T--C--------A--------T-----C--

Os fl 1306 ------------------T-----G-----------T--------------------T--C--------A--------T-----C--

ki fl 1306 ------------------C-----G-----------T--YG----------------T--Y--------A--------T-----Y--

ck hrt 1393 CATGTTCCAGCACCAGACCTCAGTTTCTCACCAACCCATTGTGCGGCAGTGTGGACCTCAGACCGGCATCCAGTCTCCCCCCAGCAG

Os hrt 1393 ---------------A-----G-----------G-----C-----------------------------------C----------A

Os fl 1393 ---------------A-----G-----------G-----C-----------------------------------C----------A

ki fl 1393 ---------A--T--A-----G-----------G-----C-----------C--------------T--------C-----------

ck hrt 1480 CTTGCAGCCTGCAGAGTTCCTCTATTCCCACGGCGTGCCTCGAACCCTCTCGCCCCACCAGTACCACTCGGTGCACGGTGTGGGCAT

os hrt 1480 ---------G-----------G-----G-----A--------------T-----------------------------C--------

os fl 1480 ---------G-----------G-----G-----A--------------T-----------------------------C--------

ki fl 1480 ---------G-----------G-----------A--------------T-----------------------------C--------

<ex9R4

ck hrt 1567 GGTGCCAGAGTGGAGCGAGAACAGC**TAA**CGAGGCAGTCGATGGAAATGGGAAAAAAAAT:AA:AACGAAATGAAAGAAAAAAGTGAA

os hrt 1567 -----------------------------A-------::-A-----:-T-C--G-GG--C--CC-T----::--TTG--G---:---

os fl 1567 -----------------------------A-------::-A-----:-T-C--G-GG--C--CC-T----::--TTG--G---:---

ki fl 1567 -----------------------------A-------::-A-----:-T-C--G-GG--C--

<ex9R

ck hrt 1652 GGGGGAAATAAGAAAAAAGGAAAGGGAAAACAAAACAAAACA:AAACAAAAAACCAGCACCCCATCAATAACAAAAACGAGAGCGTT

os hrt 1652 CAAAA---:--:-----------A------:-T-C-CT-TT-GT--T---?????????????????????????------------

os fl 1652 CAAAA---:--:-----??????????????????????????????????????????????????????????------------

ck hrt 1738 TTGCAAGTC

os hrt 1738 ---------

os fl 1738 ---------

**Figure S4**. **Sequencing strategy for moa *tbx5***. A series of overlapping sequences were obtained from a number of samples to construct moa *tbx5*. Moa sequences are compared to the exon and partial intron sequences of kiwi *tbx5*. The complete sequence was obtained from *Dinornis novaezealandiae* and *Dinornis robustus*, with additional sequence being obtained from *Megalapteryx didinus* for areas of high sequence variability (eg exon 2 and exon 8). Identical bases are shown as dashes. Gaps are indicated as colons (:). A number of moa sequences were obtained from clones that contained a number of C > T transversions (represented in lower case) that are likely to be the result of template damage. Forward primers are in blue. Reverse primers are in red. Runs of > 4 guanine or cytosine bases in primers were interupted by a thymine (t). Exon sequences are in bold capitals and intron sequences (in grey boxes) are in lower case. Sequences marked with an Ø in exon 7 are those obtained by inverse PCR on circularized moa DNA (see methods). The start codon (ATG) is shown in green and the stop codon (TAA) in red. Odd numbered coding triplets are underlined.

**ex2F> <ex2R6 CGGGGGATTCGGMGAAGGAAGCTCGTAACATGGCGGATACCGAGGAAGGCTTTGGGCTCCCG****ACCACGCCGGCTGACTCGGAGTCCAAAGAGCTGCAGGCTGA****AAGCAAGCAGGACACTC**

CM Av30495**-----------G-G------------C------------------------------G----G---------------GC--------------**

OM Av10049**-----------G-G------------C-----------------------------CG----G---------------GC--------------**

AIM B7037**-----------G-G------------C------------------------------G----G---------------GC-**

**ex2F9>**

**AGCAAGTCCtCCACGTCT <ex2R**  <i2R77 **AACTGGGGGCCACCAGCAAGTCCCCCACCTCTCCCCAGGCGGCCTTCACCCAGCAG**gtaaggacctgggcacgaatacgctccttcttctctcccccctcgctcttttttttccccttct

**-G--------------------------G-----G-----A-**

**-G--------------------------G-----G-----A-**

CM Av17563**--G-----A----------------**---::::::::::::::-cc-------c

CM Av30495**--G-----A----------------**---::::::::::::::-cc-------c--g---------:-----------c-:---

cyggrtttttyycc

ctggatttttttccc::ccaataactgttca intron2

i2F3> **ex3F>**

cgggagctgatgctttgccttcctcctttgcag**GGCATG**

MU DnTbT-----**------**

**ex3F3> <ex3R2**

**ggatcaaagtgtttttgcacga TTTCACGAAGTGGGGACC <ex3R**

**GAGGGCATAAAAGTGTTTTTGCACGAGCGGGAGCTGTGGCTGAAATTTCACGAAGTGGGGACCGAGATGATCATAACAAAGGCTGGAAG**gtaagagacgggctgaagcggtggagagcgg

**-----G--C--G---------------------------------**

**C--G-----------------------------------------G--------G** MU DgTbT

CM Av30495**---------------------------G--------G-----------**

CM Av30495**---------------------------G--------G--------------C----------**---g-------------c-------c------

AIM B7037**---------------------------G--------G--------------C----------**---g-------------c-------c------

<i3R5 agcctcctcttcccgggaggaaggcgacccacgcgctccgcgtccctctt

--------------------

---t---------------- intron3

i3F> **ex4F2>**

acacgcagccaccttcagaaactttctcttctgtgcatttatatttatgtacttttttttttttttttttatag**GCGTATGTTCCCCAGTTACAAAGTGAAGGTCACTGGACTTAATCCA**

MU DnTbT-------------ga---------------------------------------**----------------------------------------------**

MU DnTbT**---------------------------------**

**<ex4R**

**ATGGATATTGTACCAGCGGATG**

**GATATTGTACCAGCGGATGACC kx4lrF> <ex4R2** <i4R

**AAAACTAAGTACATACTGTTGATGGATATTGTACCAGCGGATGACCACAGATACAAATTTGCAGATAATAAATG**gtatgcacgcatgggggaaaggggtgggagaggagctttggatcgg

**---------------------** CM Av8317**----------------------------**------------------------c---

**------------------------------------------------------------**

CM Av30875**----------------------------**------------------------c---

intron4

**ex5F7>**

i4F> **GGTGACAGGGAAGGCAGA**

gggggccgggcggctcccggaggggtccccgcggccagctcagcgcccctgtgtccttcgcgcag**GTCGGTGACAGGGAAGGCAGAGCCGGCCATGCCCGGCCGGCTGTAC**

MU DnTbT----------**----------------------------------------------**

CM Av8317----------**----------------------------------------------**

MU DgTbT**------------------C------**

**ex5F5>**

**CCACTGGATGAGGCAGC<kx5lrR <ex5R5**

**GTCCACCCCGACTCCCCCGCCACCGGCGCCCACTGGATGAGGCAGCTGGTTTCCTTCCAAAAACTCAAGCTCACCAACAACCACCTCGACCCCTTCGGACAT**gtaagtacccgggtggga

**----------------------------------**

**----------------------------------**

**-------------------------------------------------------------**

AIM B7070**---------------------------------------------------------------------c---**

AIM B7037**---k---------------------------------------------------------------------**

<i5R33

aggggcgatgctcggygtgcgg

intron5

i5F>

tgcggggcgggggtgccgcgctgtgatccctccattcccacggggtgtcctttccttctccccgtccccyag

MU DnTbT-------g---------------a----c--

CM Av30495-------g---------------a----c--

CM Av30495-------g---------------a----c—-

**<ex6R6**

**ex6F2> CGTGAAAGCGGACGAGAACAA <ex6R5**

**ATCATCCTGAACTCC ex6F> ex6F46> TCCAAGAACACCGCCTTT ex6F4> <ex6R**

**ATCATCCTGAACTCCATGCACAAATACCAGCCCCGGCTCCACATCGTGAAAGCGGACGAGAACAACGGCTTCGGGTCCAAGAACACCGCCTTTTGCACCCACGTCTTCCCGGAGACCGCC**

**------------------t-t-----Tt----------------**

**--------------------------T-----------------**

**--------------------------T-----------------------------T--------------T---** CM Av8378**-----------**

MU DnTbT**-----------T-----------------------------T--------------T------------------------------------------------**

OM Av10049**------------------------T--------------T------------------------------------------**

MU DnTbT**------T------------------------------------------------**

**<ex6R2**

**CCTACCAAAACCACAAG** <i6R75

**TTCATCGCCGTCACCTCCTACCAAAACCACAAG**gtaaggggctgggccggccttggcaccggcaaatcgcgtttgctctccttccctccttgcacaatttcttttgaggtgcttg

-**-------------------------T-----**-----------------c----t-----a------t-t----c-----------

**----------------**

-**-------------------------T-----**---------------t-c----t-----aa-----t-t----c-----------

intron6

g**ATCACCCAGTTA**

i6F5> **GCTG**

gaactgtttagcttgggtttaatacgcagtatcctctctctcccaggccttgccttggtcgy:ctatg::cccgttccatt::ctcc::ttcag**ATCACCCAGTTA**

CM Av30495--a-tta-g---aa-----------tc----ct-----------------

CM Av30495--a-tta-g---aa-----------tc----ct-----------------

MU DnTbT--a-tta-g---aa-----------tc----ct-----------------

**<ex7R4**

**ex7F5> ex7Rrev> TCCAGGATGCAGAG**gtaacat

**AAGATTGAGA ex7F3> ex7F4> CACAGGATGTCCAGGAT** <ex7Rs

**AAGATTGAGAACAACCC ex7F2><ex7R3 CAGTGACGACATGGAGCT <ex7R AGGATGCAGAG**gtaacatg

**ex7F>GAGAACAACCCCTTTGCAAAAGGTTTCCGCGGCAGTGACG <ex7R2 CACAGGATGTCCAGGATGCA**

**AAGATTGAGAACAACCCCTTTGCGAAAGGTTTCCGCGGCAGTGACGACATGGAGCTCCACAGGATGTCCAGGATGCAGAG** <i7Rm3

**-----------------------------------T---------------------------------** Ø**------**gtaacatgtgatcctgttgtggtaacac AIM B6316

**-----------------------------------T------------------------------** Ø**------**---------------------------- CM Av30495

**-----------------------------------T------** CM Av30495**------------------------**----------Ø

**-----------------------------------T------ -----------**CM Av30495

**-----------------------------T---------------------**CM Av30495

**-------------------------T--------------------------------------------**--MU DnTbT

CM Av30495**------------------T------------------------------**

CM Av30495**----------T------**

intron7

i7F>

actgatgaagtgtggcagggctgcggtctcctgggcggatggctctatttccctgaaagtctaaacaaacaccatgacactaatgtgctgctttcatttattaattcac

CM Av30495----------------

MU DnTbT----------------

MU DnTbT----------------

**ex8F> <ex8R4 <ex8R3**

cgatctatttattaattaattgcttttctgccttsttttttcag**TAAAGAGTACCCGGTTGTTCCCAGGAGCACAGTGAGACAAAAAGTGTCCTCAAATCACAGCCCATTCAGTGGTGAG**

t-------------------------------c-c---c-----**---------------C-----------------------------------G--------------------C---**

t-------------------------------c-c---c-----**---------------C-------------------------**

t-------------------------------c-c---c-----**---------------C-------------------------**

CM Av30495**----------------G--------------------C---**

OM Av10049**----------------G--------------------C---**

**<ex8R6**

**ex8F6> <ex8R2 AGAACGGtGTGTCGAGCACYT ex8F4>**

**ACCAGGGTCCTTTCTGCCTCCTCCAACTTGGGGTCCCARTATCARTGCGAGAACGGGGTGTCGAGCACYTCCCAGGRCCTGYTRCCGCCTGCCAACCCGTACCCGATCTCSCAGGAGCAC**

**-----------C--C-----------------C**

**-----------C--C-----------------C-----a-----G----**

**-----------C--C-----------------C-----G-----G-----------------------C-------A----C-G--A-----------C-----------C---------**

CM Av30495**--C-----G-----G-----------------------C-------A----C-G--A-----------C-----------C---------**

AIM B6316**--C-----G-----G-----------------------C-------A----C-G--A-----------C-----------C---------**

AIM B7145**--C-----G-----G-----------------------C-------A----C-G--A-t---------C-----------C---------**

MU DnTbT**--C-----G-----G-----------------------C-------A----C-G--A-----------C-----------C---------**

**<ex8R**

**ACTGCACCAAGAG**

**<ex8R5** <i8R

**AGCCAGATCTACCACTGCACCAAGAGAAAAG**gtcaggccttggtggctccctgctccgctcccgctctacggctttcccattccaaacacgattgtcagtgtcgttttgtg

**------------------------------**----------------------------------------------MU DnTbT

**------------------------------**----------------------------------------------CM Av8317

**-------------**OM Av10049

**---------**

**---------**

**--------t** intron8

**---------** i8F2>

i8F> agctgagggtacggtatt

gactgagaagagtctctgcatcagctctgtgcaggctgtggccttggtaaaatgaggataatactgacagatggcagagcaggacgttcagctgagggtacggtattgt

MU DgTbT--------

CM Av17563--

MU DgTbT--

**<ex9R23**

**ex9F><ex9R7 AAGAGGATCCTTTCTACAGGT**

tatttgctaccaggatttttctctctcaacag**ATGAGGAATGTTCCACCACCGAGCATGCCTACAAGAAGCCCTACATGGAAACTTCTCCGGC****AGAAGAGGATCCTTTCTACAGGTCCAG**

--------------------------------**---------------------------**

--------------------------------**---------------------------------------------------------------**

--------------------------------**---------------------------------------------------------------**

MU DnTbT**-------------------------C-----------------------------------**

**ex9F14> <ex9R2**

**ex9F5> <ex9R3 CGCCAAGCATGTATGTA ex9F2> CCTAGAAGACATCAG**

**TTACCCCCAGCAGCAGGGACTGAACACTTCATACAGGACTGAATCAGCTCAGCGCCAAGCATGTATGTACGCCAGCTCGGCTCCCCCCACGGACCCCGTGCCCAGCCTAGAAGAYATCAG**

**------------------------------------------------------G--------------------A-------------------------------**CM Av30495

**------------------------------------------------------G--------------------A-----------------------**CM Av30495

**------------------------------------------------------G--------------------A-----------------------**MU DnTbT

**----------------** CM Av17563-------**-----A--------------------------------C-----**

MU DnTbT**--------A------------------------t-------C-----**

**ex9F11>**

**CCATGCAGCCGATGGACAGGT <ex9R24**

**CT <ex9R19 ex9F3> TTACCCTACCAGCATTTCTCT <ex9R5**

**CTGTAACACGTGGCCGAGCGTGCCCTCCTACAGCAGTTGCACAGTATCTGCCATGCAGCCGATGGACAGGTTACCCTACCAGCATTTCTCTGCCCACTTCRCCTCCGGGCCGCTGATGCC**

**---------------C--------------------C-----G--G------------------------**

**---------------C--------------------C-----G--G----------------------------**

CM Av30495**----------------------------------T------A-------------------**

MU DnTbT**----------------------------------T------A-------------------**

AIM B7037**----------------------T------A-------------------**

**ex9F9>**

**GGCAGCGTGGCCAACCATAC**

**ex9F7> <ex9R9 <ex9R20**

**CCGTCTYGGCAGCGTGGCCAACCATACYTCCCCCCAAATAGGAGATACCCAYAGCATGTTCCAACATCAAACCTCGGTTTCTCACCAGCCCATCGTGCGGCAGTGCGGACCTCAGACCGG**

**------G--------------------C----------**

**------G--------------------C----------**

**------G--------------------C-----------------C-----C--------------C—**

AIM B7037**C---t-------------C-----C--------------C-----------------------------------------------------**

AIM B6316**C-----------------C-----C--------------C-----------------------------------------------------**

CM Av30495**C------t----------C-----C--------------C-----------------------------------------------------**

CM Av30495**--------------C-----C--------------C-----------------------------------------------------**

**<ex9R14**

**<ex9R11 acggagtgcctcgaaccctttcg**

**ex9F8> ex9F4> AGAGTTCCTGTATTCCCAC**

**TATCCAGTCCCCCCCCAGCAGCTTGCAGCCGGCAGAGTTCCTGTATTCCCACGGAGTGCCTCGAACCCTTTCGCCCCACCAGTATCACTCGGTGCACGGCGTGGGCATGGTGCCAGAGTG**

**C---t------t--------------------------------------**

**C-------------------------------------------------**

**C---------------------------------a---------------**

**C--------------------------------**

AIM B6316**----------------------------------------------------------------------------C-----------------------------------**

AIM B7072**---------------------------------------------C-----------------------------------**

AIM B7145**---------------------------------------------C-----------------------------------**

CM Av30495**---------------------------------------------C-----------------------------------**

CM Av9032**---------------------------------------------C-----------------------------------**

**<ex9R13**

**AGAGGATCAGCCATGAAAAATTGAG**

**GAGCGAGAACAGCTAACAAGG****CAGTAAGGAGAGTGCGAGAGGATCAGCCATGAAAAATTGAGGAAAAAAAA**

**------------------------C-----A------**

**------------------------C-----A------**

**------------------------C-----A------**

**------------------------C-----A------**

**------------------------C--a--A------**

**Figure S5**. ***tbx5* exon 2 clones for *Dinornis novaezealandiae* (AIM B7037)**. PCR products produced using primers ex2F / ex2R6 were cloned into vector pUC19 and sequenced with m13R. 24 clone sequences were aligned to determine levels of DNA template damage. 5 sequence variants were detected. As expected most damage resulted from C > T transitions. A single T > C transition is present on two different clones (9 and 22) and may be the result of heterozygosity that would result in an aromatic phenylalanine (F; in grey) or a nucleophilic serine (S) at amino acid position 8. The consensus sequence matches that obtained from direct PCR product sequencing.

AIM B7037_17.m13R **------------------------------------------------------------------y--------------**

AIM B7037_13.m13R **---------------------------------------------------------------------------------**

AIM B7037_11.m13R **---------------------------------------------------------------------------------**

AIM B7037_23.m13R **---------------------------------------------------------------------------------**

AIM B7037_6.m13R **---------------------------------------------------------------------------------**

AIM B7037_27.m13R **---------------------------------------------------------------------------------**

AIM B7037_31.m13R **---------------------------------------------------------------------------------**

AIM B7037_32.m13R **---------------------------------------------------------------------------------**

AIM B7037_25.m13R **---------------------------------------------------------------------------------**

AIM B7037_4.m13R **---------------------------------------------------------------------------------**

AIM B7037_14.m13R **---------------------------------------------------------------------------------**

AIM B7037_19.m13R **---------------------------------------------------------------------------------**

AIM B7037_26.m13R **---------------------------------------------------------------------------------**

AIM B7037_3.m13R **---------------------------------------------------------------------t-----------**

AIM B7037_24.m13R **---------------------------------------------------------------------t-----------**

AIM B7037_15.m13R **---------------------------------------------------------------------t-----------**

AIM B7037_12.m13R **---------------------------------------------------------------------t-----------**

AIM B7037_10.m13R **---------------------------------------------------------------------t-----------**

AIM B7037_9.m13R **-------------------------c-------------------------------------------------------**

AIM B7037_22.m13R **-------------------------c-----------------t------t-t----------------------------**

AIM B7037_30.m13R **-------------------------------------------t------t-t----------------------------**

AIM B7037_18.m13R **-------------------------------------------t------t-t----------------------------**

AIM B7037_8.m13R **-------------------------------------------t------t-y----------------------------**

AIM B7037_1.m13R **-------------------------------------------t------t-t----------------------------**

Consensus **AACATGGCGGATACCGAGGAAGGCTTTGGGCTCCCGACCACGCCGGCTGACTCGGAGTCCAAAGAGCTGCAGGCTGAAAGC**

Amino acid  M A D T E E G **F** G L P T T P A D S E S K E L Q A E S

**Figure S6**. ***tbx5* amino acid sequence lineup for chicken, kiwi, ostrich, and *Dinornis***. Amino acid changes are in red boxes. The T-box DNA binding region is shown in green. Nuclear localisation signals (NLS) are shown in khaki (Collavoli et al, 2003) and a nuclear export signal (NES) in grey (Kulisz and Simon, 2008). Both NLS sequences are required for nuclear localisation. The region required for transcriptional transactivation is shown in blue (Zaragoza et al, 2004). Most variation is seen in the NH2 region before the Tbox motif. This region has been shown to be important for binding to Tbx5’s transcriptional activation partner NKX2.5 and subsequent activation of downstream targets atrial natriuretic factor (ANF) and Connexin 40 (Cx40). In addition two missense mutations, Q49K and I54T, identified in HOS patients, have been shown to inhibit Tbx5 binding to Sall4 thereby reducing the transcriptional activation of *fgf10* (Koshiba-Takeuchi et al, 2005). Furthermore the carboxy (COOH) terminus of Tbx5 (3’ of the Tbox) has been shown to bind the WW domain containing proteins TAZ and YAP, also important for *fgf10* activation (Murakami et al, 2005).

chk MADTEEGFGLPSTPVDSEAKELQAEAKQDPQLGTTSKAPTSPQAAFTQQGMEGIKVFLHERELWLKFHEVGTEMIITKAGRRMF 84

kiw MADTEEGFGLPTTPADSESKELQAESKQDTQLGATSKSPTSPQAAFTQQGMEGIKVFLHERELWLKFHEVGTEMIITKAGRRMF 84

ost MADTEEGFGLPTTPADSESKELQAETKQDTQLGATSKSPTSPQAAFTQQGMEGIKVFLHERELWLKFHEVGTEMIITKAGRRMF 84

Dns MAESEEGFGLPTTPADSEAKELQAEAKQDTQLGATSKSPTSPQAAFTQQGMEGIKVFLHERELWLKFHEVGTEMIITKAGRRMF 84

chk PSYKVKVTGLNPKTKYILLMDIVPADDHRYKFADNKWSVTGKAEPAMPGRLYVHPDSPATGAHWMRQLVSFQKLKLTNNHLDPF 168

kiw PSYKVKVTGLNPKTKYILLMDIVPADDHRYKFADNKWSVTGKAEPAMPGRLYVHPDSPATGAHWMRQLVSFQKLKLTNNHLDPF 168

ost PSYKVKVTGLNPKTKYILLMDIVPADDHRYKFADNKWSVTGKAEPAMPGRLYVHPDSPATGAHWMRQLVSFQKLKLTNNHLDPF 168

Dns PSYKVKVTGLNPKTKYILLMDIVPADDHRYKFADNKWSVTGKAEPAMPGRLYVHPDSPATGAHWMRQLVSFQKLKLTNNHLDPF 168

chk GHIILNSMHKYQPRLHIVKADENNGFGSKNTAFCTHVFPETAFIAVTSYQNHKITQLKIENNPFAKGFRGSDDMELHRMSRMQS 252

kiw GHIILNSMHKYQPRLHIVKADENNGFGSKNTAFCTHVFPETAFIAVTSYQNHKITQLKIENNPFAKGFRGSDDMELHRMSRMQS 252

ost GHIILNSMHKYQPRLHIVKADENNGFGSKNTAFCTHVFPETAFIAVTSYQNHKITQLKIENNPFAKGFRGSDDMELHRMSRMQS 252

Dns GHIILNSMHKYQPRLHIVKADENNGFGSKNTAFCTHVFPETAFIAVTSYQNHKITQLKIENNPFAKGFRGSDDMELHRMSRMQS 252

chk KEYPVVPRSTVRQKVSSNHSPFSGETRVLSTSSNLGSQYQCENGVSSTSQDLLPPTNPYPISQEHSQIYHCTKRKDEECSTTEH 336

kiw KEYPVVPRSTVRQKVSSNHSPFSGETRVLSASSNLGSQYQCENGVSSTSQGLLPPANPYPISQEHSQIYHCTKRKDKECSTTEH 336

ost KEYPVVPRSTVRQKVSSNHSPFSGETRVLSASSNLGSQYQCENGVSSTSQDLLPPANPYPISQEHSQIYHCTKRKDEECSTTEH 336

Dns KEYPVVPRSTVRQKVSSNHSPFSGETRVLSASSNLGSQYQCENGVSSTSQDLLPPANPYPISQEHSQIYHCTKRKDKECSTTEH 336

chk PYKKPYMETSPAEEDPFYRSSYPQQQGLNTSYRTESAQRQACMYASSAPPTDPVPSLEDISCNTWPSVPSYSSCTVSAMQPMDR 420

kiw AYKKPYMETSPAEEDPFYRSSYPQQQGLNTSYRTESAQRQACMYASSAPPTDPVPSLEDISCNTWPSVPSYSSCTVSAMQPMDR 420

ost AYKKPYMETSPAEEDPFYRSSYPQQQGLNTSYRTESAQRQACMYASSAPPTDPVPSLEDISCNTWPSVPSYSSCTVSAMQPMDR 420

Dns AYKKPYMETSPAEEDPFYRSSYPQQQGLNTSYRTESAQRQACMYASSAPPTDPVPSLEDISCNTWPSVPSYSSCTVSAMQPMDR 420

chk LPYQHFSAHFTSGPLMPRLSSVANHTSPQIGDTHSMFQHQTSVSHQPIVRQCGPQTGIQSPPSSLQPAEFLYSHGVPRTLSPHQ 504

kiw LPYQHFSAHFTSGPLMPRLGSVANHTSPQIGDTHSMFQHQTSVSHQPIVRQCGPQTGIQSPPSSLQPAEFLYSHGVPRTLSPHQ 504

ost LPYQHFSAHFTSGPLMPRLSSVANHTSPQIGDTHSMFQHQTSVSHQPIVRQCGPQTGIQSPPSNLQPAEFLYSHGVPRTLSPHQ 504

Dns LPYQHFSAHFTSGPLMPRLGSVANHTSPQIGDTHSMFQHQTSVSHQPIVRQCGPQTGIQSPPSSLQPAEFLYSHGVPRTLSPHQ 504

chk YHSVHGVGMVPEWSENS. 521

kiw YHSVHGVGMVPEWSENS. 521

ost YHSVHGVGMVPEWSENS. 521

Dns YHSVHGVGMVPEWSENS. 521

**Figure S7**. ***tbx5* amino acid sequence lineup of the NH2 terminus.** The NH2 terminal 60 amino acids of moa were compared to the translated NCBI database. Amino acid changes that differ from the consensus are in red boxes. Known mutations that disrupt Sall4 binding (Q49K and I54T) are shown at the top in bold (Koshiba-Takeuchi et al, 2005). A single amino acid (E; glutamic acid) at the highly conserved position three is unique to moa. Dns - Dinornis, zbf - zebra finch, trk - turkey, chk - chicken, enw - eastern newt, xnp - xenopus, ops - opossum, hum - human, plp - platypus, elp - elephant, mse - mouse, zfs - zebrafish.

**K_49_ T_54_**

Dns MAESEEGFGLPTTPADSEAKELQAEAKQDTQLGATSKSPTSPQAAFTQQGMEGIKVFLHE 60

kiw MADTEEGFGLPTTPADSESKELQAESKQDTQLGATSKSPTSPQAAFTQQGMEGIKVFLHE 60

ost MADTEEGFGLPTTPADSESKELQAETKQDTQLGATSKSPTSPQAAFTQQGMEGIKVFLHE 60

[zbf](http://www.ncbi.nlm.nih.gov/nucleotide/224071923?report=genbank&log$=nuclalign&blast_rank=1&RID=5CXBH4CV014) MADGEEGFGLPGTPADSEAKELQAEGKQDTQLGATSKSPTSPQAAFTQQGMEGIKVFLHE 60

[trk](http://www.ncbi.nlm.nih.gov/nucleotide/326930079?report=genbank&log$=nuclalign&blast_rank=2&RID=5CXBH4CV014) MADTEEGFGLPSTPADSEAKELQAEAKQDPQLGTTSKAPTSPQAAFTQQGMEGIKVFLHE 60

chk MADTEEGFGLPSTPVDSEAKELQAEAKQDPQLGTTSKAPTSPQAAFTQQGMEGIKVFLHE 60

enw MADSDEGFGMPDTPVDPESKELQSDSKQDSQLGAGSKPPSSPQAAFTQQGMEGIKVFLHE 60

[xn](http://www.ncbi.nlm.nih.gov/nucleotide/73665909?report=genbank&log$=nuclalign&blast_rank=7&RID=5CXBH4CV014)p MADTEEAYGMPDTPVEAEPKELQCEPKQDNQMGASSKTPTSPQAAFTQQGMEGIKVFLHE 60

[ops](http://www.ncbi.nlm.nih.gov/nucleotide/334327242?report=genbank&log$=nuclalign&blast_rank=10&RID=5CXBH4CV014) MADADEAFGLPHTPLEAESKELPPEAKQENPLGSSSKAPASPQAAFTQQGMEGIKVFLHE 60

[hum](http://www.ncbi.nlm.nih.gov/nucleotide/2281318?report=genbank&log$=nuclalign&blast_rank=11&RID=5CXBH4CV014) MADADEGFGLAHTPLEPDAKDLPCDSKPESALGAPSKSPSSPQAAFTQQGMEGIKVFLHE 60

[plp](http://www.ncbi.nlm.nih.gov/nucleotide/345314777?report=genbank&log$=nuclalign&blast_rank=12&RID=5CXBH4CV014) MADAEDGFDVSHTPLDPDVKELASEAKAENPLGTSGKSPGSPQAAFTQQGMEGIKVFLHE 60

[elp](http://www.ncbi.nlm.nih.gov/nucleotide/344295255?report=genbank&log$=nuclalign&blast_rank=13&RID=5CXBH4CV014) MADADEGFGLAHTPLEPESKDLPCDSKPESTLGAASKSPSSPQAAFTQQGMEGIKVFLHE 60

dog MADADEGFGLAHTPLEPDSKDLPCDSKAESSLGAPSKSPASPQAAFTQQGMEGIKVFLHE 60

[pig](http://www.ncbi.nlm.nih.gov/nucleotide/311270690?report=genbank&log$=nuclalign&blast_rank=36&RID=5CXBH4CV014) MADGDEGFGLAHTPLEPDSKDLPCDSKPESGLGAPSKSPSSPQAAFTQQGMEGIKVFLHE 60

[mse](http://www.ncbi.nlm.nih.gov/nucleotide/6002731?report=genbank&log$=nuclalign&blast_rank=42&RID=5CXBH4CV014) MADTDEGFGLARTPLEPDSKDRSCDSKPESALGAPSKSPSSPQAAFTQQGMEGIKVFLHE 60

[zfs](http://www.ncbi.nlm.nih.gov/nucleotide/7328572?report=genbank&log$=nuclalign&blast_rank=52&RID=5CXBH4CV014) MADSEDTFRLQNSPSDSEPKDLQNEGKSDKQNAAVSKSPSS-QTTYIQQGMEGIKVYLHE 60

**Figure S8**. ***tbx5* intron-exon boundary sequences**. Consensus donor and acceptor splice sites (and position numbers) are shown (Con; Zhang, 1998). Coding sequences are in capitals, intron sequences are in lowercase lettering. hum - human, mse - mouse, chk - chicken, kiw - kiwi, Dns - *Dinornis*. 2 - 8 refer to intron number. Bases in red represent conserved intron donor (5’ gt) and acceptor (3’ ag) sequences. Gaps are shown by dashes. A single intervening sequence change from the consensus G to A at position 5 (IVS2 + 5G > A) in *Dinornis tbx5* intron 2 (shaded box) has been shown by others to result in either retention of the affected intron in the mRNA (Asselta et al, 2000 ) for the human fibrinogen gamma gene (FGG), or deletion of the preceeding exon (Margaglione et al, 2000). This sequence change however, is unlikely to have an affect on moa, as this splice site is highly conserved with that from tinamou.

Donor+12345 321-Acceptor

Con **-------AG**gtaagt---------------------------cag**G-----**

hum2 **GCCTTCACCCAGCAG**gtaaggagacctcgc------ttctccttcttgcag**GGCATGGAGGGAATC**

mse2 **GCCTTCACCCAGCAG**gtaagaaaagccggc------tctttgtctatcaag**GGCATGGAAGGAATC**

chk2 **GCCTTCACCCAGCAG**gtaaggagcggaccg------cttcctcctttgcag**GGCATGGAGGGGATC**

kiw2 **GCCTTCACCCAGCAG**gtaaggacctgggca------cttcctcctttgcag**GGCATGGAGGGCATA**

Dns2 **GCCTTCACCCAGCAG**gtaaacccgctcctc----------------tgcag**GGCATGGAGGGGATC**

hum3 **AACCAAGGCTGGAAG**gtgagatggtttgtt------gtccctctctcttag**GCGGATGTTTCCCAG**

mse3 **CACCAAGGCAGGGAG**gtgagccagctcctg------tttctttttcctcag**GAGAATGTTTCCTAG**

chk3 **AACAAAGGCTGGAAG**gtaagaagcagcccc------ttctttcttttatag**GCGTATGTTTCCCAG**

kiw3 **AACAAAGGCTGGAAG**gtaagagacgggctg------tttttttttttatag**GCGTATGTTCCCCAG**

Dns3 **AACCAAGGCTGGAAG**gtgagagacgggccg------tttttttttttatag**GCGTATGTTCCCCAG**

hum4 **CGCAGATAATAAATG**gtaggcactggggtg------ctctccttcatctag**GTCTGTGACGGGCAA**

mse4 **TGCTGATAACAAATG**gtaggttccagggtt------ttctccttcatgtag**GTCCGTAACTGGCAA**

chk4 **TGCAGATAATAAATG**gtacgcacgccgggg------ctctgtcccacgcag**GTCCGTGACCGGGAA**

kiw4 **TGCAGATAATAAATG**gtatgcacgcatggg------gtgtccttcgcgcag**GTCGGTGACAGGGAA**

Dns4 **TGCAGATAATAAATG**gtatgcacgcatggg--------------cgcgcag**GTCGRTGACAGGGAA**

hum5 **GACCCATTTGGGCAT**gtgagtaccgtggcc------ctttattatttttag**ATTATTCTAAATTCC**

mse5 **GACCCGTTTGGACAC**gtaagtaccctgtct------ctctgttatttttag**ATTATCCTGAACTCC**

chk5 **GACCCCTTCGGACAT**gtgagtaccgggctg------tctccccatgcccag**ATCATCCTGAACTCC**

kiw5 **GACCCCTTCGGACAT**gtaagtacccgggtg------ctccccgtccccyag**ATCATCCTGAACTCC**

Dns5 **GACCCCTTCGGACAT**gtaagtacccgggcg------ctccccgaccccyag**ATCATCCTGAACTCC**

hum6 **TACCAGAACCACAAG**gtaagcctgaagccc------tcctctttccttcag**ATCACGCAATTAAAG**

mse6 **TACCAGAATCACAAG**gtaagcctgagagag------ctccttctctctcag**ATCACACAGCTGAAA**

chk6 **TACCAAAACCACAAG**gtgagggctgggccg------tttcctccctttcag**ATCACTCAGCTGAAG**

kiw6 **TACCAAAACCACAAG**gtaaggggctgggcc------tccattctccttcag**ATCACCCAGTTAAAG**

Dns6 **TACCAAAACCACAAG**gtaaggggctgggcc------tttcctccctttcag**ATCACCCAGTTAAAG**

hum7 **GTCAAGAATGCAAAG**gtaggaaagtggatt------tcttttctctttcag**TAAAGAATATCCCGT**

mse7 **GTCTCGGATGCAAAG**gtaagaaatcggggc------tcttcttcctttcag**TAAAGAGTATCCTGT**

chk7 **GTCCAGGATGCAGAG**gtaatgcatgcatcc------tttgtttgcttttag**TAAAGAGTACCCAGT**

kiw7 **TACCAAAACCACAAG**gtaaggggctgggcc------gccttgttttttcag**TAAAGAGTACCCGGT**

Dns7 **TACCAAAATCACAAG**gtaaggggctgggct------gccctctttcttcag**TAAAGAGTACCCGGT**

hum8 **GTACCAAGAGGAAAG**gtgagtgtgatcacc------ctcctgtcttcacag**AGGAAGAATGTTCCA**

mse8 **GTACCAAGAGGAAAG**gtgagtgtggcaggc------ttcctgtctttgcag**ATGAGGAATGTTCCA**

chk8 **GCACCAAGAGAAAAG**gtcaggccttcaata------tttctctcccagcag**ATGAGGAATGTTCCA**

kiw8 **GCACCAAGAGAAAAG**gtcaggccttggtgg------tttctctctcaacag**ATAAGGAATGTTCCA**

Dns8 **GCACCAAGAGAAAAG**gtcaggccttggtgg------tttctctctcaacag**ATAAGGAATGTTCCA**


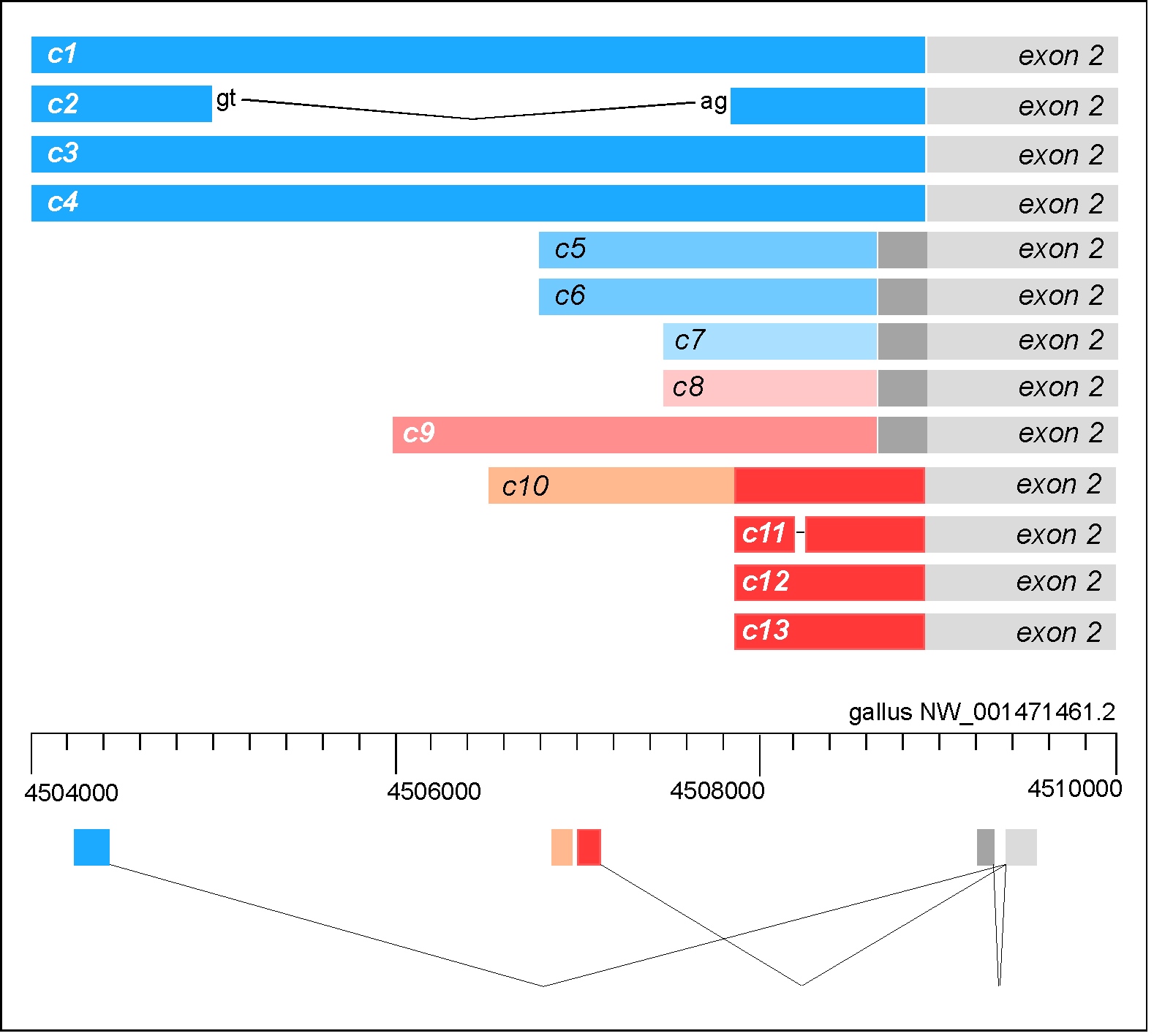


**Figure S9. Ostrich forelimb and heart *tbx5* exon 1 cDNA sequences**. Approximately 5ug of early ostrich embryo forelimb and heart RNA was reverse transcribed into cDNA as described and tailed with dATP (Methods). Nested 5’ RACE (Rapid Amplification of cDNA Ends) was then carried out using H5FdT (5’- AATCGGACAAACTGGTCCTTGCAACdT_20_) and ex2R2 (5’- GGTGAGCGACTTGCTGGTG), followed by H5F (5’- AATCGGACAAACTGGTCCTTGCAAC) and ex2R3 (5’- CAAAGCCTTCCTCCGTAT). Amplified products were TA cloned into pGEM®T-Easy (Promega) and sequenced with m13F (5’-TGTAAAACGACGGCCAGT) or m13R (5’-CAGGAAACAGCTATGACC). Thirteen clones (c1 - c13) representing all variants detected are shown (top, not to scale). Light grey boxes represent exon 2 sequences. Blue boxes are exon 1 sequences obtained from embryonic ostrich forelimb cDNA. Red boxes are exon 1 sequences from embryonic ostrich heart cDNA. Sequences represented by the dark grey box were found in *tbx5* cDNAs from both heart and forelimb. Comparison with the chicken genome (Build 3.1) positioned the forelimb-specific exon 1 approximately 5 kb upstream from exon 2 and the heart-specific exon 1 approximately 2.5 kb upstream from exon 2 (bottom). No significant homology was found to chicken for ostrich exon 1 sequences from clones c5-c9. A deletion was found in clone c2 that may correspond to an internal intron as the termini of the deleted sequence harbours consensus donor (gt) and acceptor (ag) splice sites. Comparison of the ostrich exon 1 sequences with *tbx5* cDNAs on NCBI GenBank showed that clones c1-c4 shared homology with mRNAs from *Homo sapiens* (transcript variants 1 and 3). Both these variants (variant 1 - NM_000192.3 and variant 3 - NM_080717.2) were constructed from sequences obtained from pooled lung, spleen, placental, and foetal mRNA.

emu -------------------::::--------------------------------------------------------------

cass -------------------::::--------------------------------------------------------------

kiwi -------------------------------------------------------------------------------------

ostr -------------------------------------------------------------------------------------

rhea -------------------------------------------------------------------------------------

tin ----------------------------------------T--------------------------------------------

Dn -------------------------------------------------------------------------------------

cons AGCTATCGCCTTGAACTCTCTTTATTTTATTGGAGTATGGCTGGTAATAAACAGTAATATTTAATTTGTCTGAGACCACAAATCG 90

ex1F2> <ex1R1

emu -----------------------------G-------------G-----------------------C------------:::::

cass --------------------------C--G----C--------------------------------C------------:::::

kiwi --------------------------------------------------------------------------------:::::

ostr -C-------------A-------------------------T-----------------C-------------------------

rhea ------------------------------------G----------------------------C-------------C---C-

tin ----------------T---C------------------------G----------------------C------Y---------

Dn ----------------------------------G---------------------------------C----------------

cons GTTTCTAGCTGGAAGGCTCCTTCGCCTTGACATATACAGTCCTAGAGAGCCTGGACTTGGGGTCCTTTTCCCAGCTTTT:TTTTT 180

ex1F3> <ex1R2

emu ::::::::::::::::::::--C-T--TC----A-T---A---------------------------------------------

cass ::::::::::::::::::::----TC-TC----G-T---A-----G---------------------------------------

kiwi ::::::::::::::------CC-CC-TG-T-CA---C-----------------------G----------G-------------

ostr ------::::::::AA--CC--G-TCA-C---C---C-----T----------------------------G-------------

rhea --C---------------CC-:::C----G-G-T--A--T---------------------------------------------

tin ------------------------TAA----C---G-T--A----T---------------------C----------C------

Dn -----CY-::::-----------CC-----G---A---T----T---------------:----------------G--------

cons TTTTTTTTTTTTTTTTTTTTTTTTYTCCTC:TTC:CTC:CCCCCCCCAACCTGCAGACGGAAATAAATTCGATTTATTTGCATCG 270

OexF1> exF4> <ex1R3

emu ---C----------------------C-------------G---------------------C-----------G----C-C--G

cass ---C----------------------C-------------GTC-G-----------------C-----------G------C:-G

kiwi --------------------C--C--G--------------T---------------------T---A-----------------

ostr ---------------------------------------------------------------T-------T-------------

rhea ----------C----------------------------C-Y--------------T------T---A---T-A---------T-

tin -----T--C--------------G----C-----CC---C-----G--T--------:::::::::---:T--A-------C-G-

Dn -----G-----------------G----------CC---C-----------------------------G---------------

cons TTTTCAGCTTGTCTTCAAGGTGTTTGAGAGCTAGTTTGGAACTGAAGAGgtgagtgcttccttcgcagcagcagagctttctgaa 360

OexF2> ex1F5>

emu -----------------C-G--CC--GG-----G---------G-C--CC-C---------------

cass -----------------CCG--CC--GG-----G---------C-G---C-G---------------

kiwi ----T------T-------------------------------G-----------------------

ostr --C-T------T------------T--A---------------------------------------

rhea ----T------T-----------G---------C-----------------C---------------

tin --G----:---------C-C-C-C--C----G----------A------------------------

Dn --------------------------C----G----------------------T------------

cons gcagcgggcagcagccgtgtttaacgttcgctgtggcaactt:agagattttcacttttgcctttct 427

<ex1R4

**Figure S10. Comparison of ratite forelimb *tbx5* exon 1 sequences**. ‘Full-length’ forelimb exon 1 sequences were obtained for ostrich using primers designed to upstream regions of homologous chicken sequences. These primers were used (with primer ex2R2) to amplify cDNA from ostrich forelimb. The exon 1 / intron 1 boundary was obtained by making use of the CG rich area common to the 5’ terminus of all introns. To bind to this area, a primer was designed, AnchdC (5’- GCTCGATCCTAGGATCGAGC_12_) and used in a nested PCR with the ostrich forelimb specific exon 1 primers Oex1F (5’- AACCTGCAGACGGAAAT) and Oex1F2 (5’- TCGGTTTATTTGCATCGTT), marked in blue in the ostrich sequence, to amplify the ostrich exon 1 / intron 1 boundary. Using the chicken primer ckflpF (5’- ACCTTCCATTACTGCTGCA) and a conserved intron primer ex1R (5’-CCTCGCCAGAAAGAAAGGCAAA) approximately 350 bp of exon 1 was recovered for all extant ratites. Conserved primers were then designed to amplify the homologous region from *Dinornis* (samples AIM B7037 and AIM B6316). Forward primers are shown in blue, reverse primers are shown in red. cass - cassowary, ostr - ostrich, tin - tinamou major, Dn - *Dinornis*, cons - consensus sequence. Intron sequence is in lower case. For sequence analysis, the shaded areas (including intron sequences and a TC rich area difficult to align) were removed.

Emu emu

Cas 0.021 cas

Kiw 0.042 0.055 kiw

Ost 0.047 0.060 0.033 ost

Rhe 0.042 0.056 0.038 0.038 rhe

Tin 0.097 0.111 0.087 0.092 0.078 tin

Dnr 0.065 0.074 0.056 0.060 0.047 0.060

**Figure S11. Pairwise distance comparison of ratite forelimb *tbx5* exon 1 sequences**. Evolutionary divergence was determined between sequences using MEGA 5.05 (Tamura et al, 2011). Analyses were conducted using the Maximum Composite Likelihood model (Tamura et al, 2004).


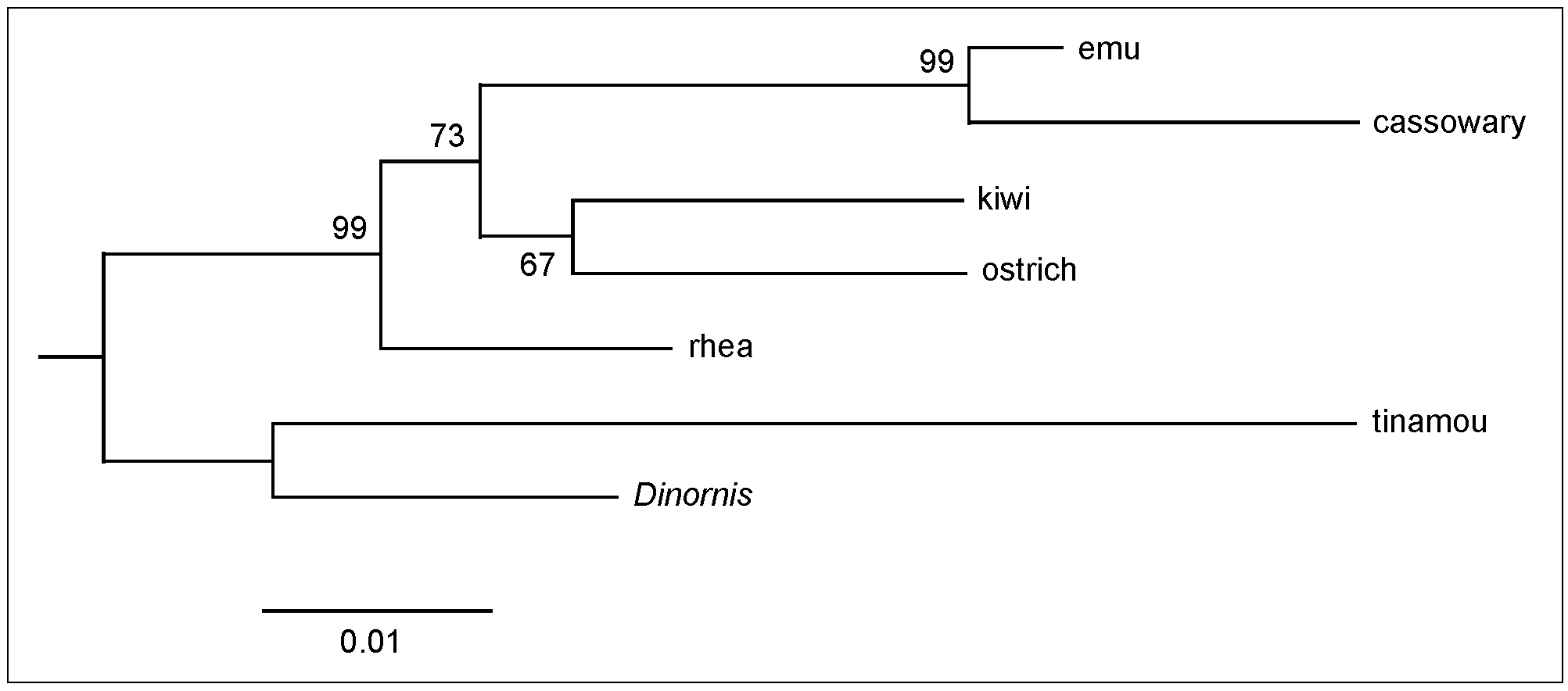


**Figure S12. Phylogenetic analysis of ratite *tbx5* exon 1 using the Maximum Likelihood method.** Trees were constructed in MEGA5.05 using the Tamura-Nei model (Tamura and Nei, 1993). The tree with the highest log likelihood (-576.73) is shown. Bootstrap values for 500 replicates are shown.

**References**

Asselta R, Duga S, Simonic T, et al. (2000) Aﬁbrinogenemia: ﬁrst identiﬁcation of a splicing mutation in the ﬁbrinogen gamma chain gene leading to a major gamma chain truncation. Blood. 96, 2496-2500.

Collavoli A, Hatcher CJ, He J, Okin D, Deo R, and Basson CT (2003) TBX5 nuclear localization is mediated by dual cooperative intramolecular signals. J Mol Cell Cardiology 35, 1191-1195.

Cooper A and Poinar HN (2000). Ancient DNA: Do it right or not at all. Science 289, 1139.

Fan C, Liu M, and Wang Q (2003). Functional analysis of Tbx5 missense mutations associated with Holt-Oram syndrome. J Biol Chem 278: 8780-8785.

Ghosh TK, Packham EA, Boser AJ, Robinson TE, Cross SJ, and Brook JD (2001). Characterization of the Tbx5 binding site and analysis of mutations that cause Holt-Oram syndrome. Hum Mol Genet 10: 1983-1994.

Huynen L, Millar CD, Scofield RP, and Lambert DM (2003). Nuclear DNA sequences detect species limits in ancient moa. Nature 425: 175-178.

Isaac A, Rodriguez-Esteban C, Ryan A, Altabef M, Tsukui T, Patel K, Tickle C, and Izpisua-Belmonte JC (1998) Tbx genes and limb identity in chick embryo development. Development 125, 1867-1875.

Kulisz A and Simon HG (2008) An evolutionarily conserved nuclear export signal facilitates cytoplasmic localization of the Tbx5 transcription factor. Mol and Cell Biol. 28, 1553-1564.

Margaglione M, Santacroce R, Colaizzo D, et al. (2000) A G-to-A mutation in IVS-3 of the human gamma ﬁbrinogen gene causing aﬁbrinogenemia due to abnormal RNA splicing. Blood. 96, 2501-2505.

Sambrook J and Russell DW (2001) Molecular Cloning, Volume 3, 3^rd^ edition. Cold Spring Harbor Laboratory Press, Cold Spring Harbor, NY, USA.

[Suzuki T](http://www.ncbi.nlm.nih.gov/pubmed?term=Suzuki%20T%5BAuthor%5D&cauthor=true&cauthor_uid=18334652), [Hasso SM](http://www.ncbi.nlm.nih.gov/pubmed?term=Hasso%20SM%5BAuthor%5D&cauthor=true&cauthor_uid=18334652), and [Fallon JF](http://www.ncbi.nlm.nih.gov/pubmed?term=Fallon%20JF%5BAuthor%5D&cauthor=true&cauthor_uid=18334652). (2008) Unique SMAD1/5/8 activity at the phalanx-forming region determines digit identity. Proc Natl Acad Sci U S A 105: 4185-4190.

Suzuki T and Ogura T. (2008) [Congenic method in the chick limb buds by electroporation.](http://www.ncbi.nlm.nih.gov/pubmed/18638168) Dev Growth Differ. 50: 459-465.

Tamura K, and Nei M (1993). Estimation of the number of nucleotide substitutions in the control region of mitochondrial DNA in humans and chimpanzees. Mol Biol and Evol 10, 512-526.

Tamura K, Nei M, and Kumar S, (2004). Prospects for inferring very large phylogenies by using the neighbour-joining method. Proc Natl Acad Sci USA 101, 11030-11035.

Tamura K, Peterson D, Peterson N, Stecher G, Nei M, and Kumasr S (2011). MEGA5: Molecular evolutionary genetics analysis using maximum likelihood, evolutionary distance, and maximum parsimony methods. Mol Biol and Evol

Zaragoza MV, Lewis LE, Sun G, Wang E, Li L, Said-Salman I, Feucht L, Huang T (2004) Identification of the TBX5 transactivating domain and the nuclear localization signal. Gene 330, 9-18.

Zhang MQ, (1998) Statistical features of human exons and their flanking regions. Hum Mol. Genet. 7, 919-932.
